# Virtual Cocrystal Screening of Adefovir Dipivoxyl: Identification of New Solid Forms with Improved Dissolution and Permeation Profiles

**DOI:** 10.3390/pharmaceutics14112310

**Published:** 2022-10-27

**Authors:** Rafael Barbas, Hanan Fael, Samuel Lee, Rebeca Ruiz, Christopher A. Hunter, Elisabet Fuguet, Clara Ràfols, Rafel Prohens

**Affiliations:** 1 Unitat de Polimorfisme i Calorimetria, Centres Científics i Tecnològics, Universitat de Barcelona, Baldiri Reixac 10, 08028 Barcelona, Spain; 2 Departament d’Enginyeria Química i Química Analítica, Universitat de Barcelona, Martí i Franquès 1-11, 08028 Barcelona, Spain; 3 Pion Inc., Forest Row Business Park, Forest Row, East Sussex RH18 5DW, UK; 4 Yusuf Hamied Department of Chemistry, University of Cambridge, Cambridge CB2 1EW, UK; 5 Serra Húnter Programme, Generalitat de Catalunya, 08017 Barcelona, Spain

**Keywords:** Adefovir Dipivoxyl, polyphenols, cocrystal, cocrystallization, computational screening, dissolution rate, permeability

## Abstract

The application of a computational screening methodology based on the calculation of intermolecular interaction energies has guided the discovery of new multicomponent solid forms of the oral antiviral Adefovir Dipivoxyl. Three new cocrystals with resorcinol, orcinol and hydroquinone have been synthesized and thoroughly characterized. They show improved dissolution profiles with respect to the single solid form, particularly the cocrystals of orcinol and resorcinol, which have 3.2- and 2-fold faster dissolution rates at stomach conditions (pH 1.5). Moreover, dynamic dissolution experiments that simultaneously mimic both the pH variation along the gastrointestinal tract and the partition into biological membranes show that, in addition to the faster initial dissolution, Adefovir Dipivoxyl also penetrates faster into the organic membranes in the form of resorcinol and orcinol cocrystals.

## 1. Introduction

The formulation of pharmaceutical drugs in the form of cocrystals has experienced important advances in the last decade through the discovery of thousands of cocrystals, [1,2] together with the generation of a huge corpus of experimental data and the development of theoretical approaches for their study [3,4,5]. This includes a deep knowledge about the intermolecular forces involved in their formation [6,7], which has allowed the design of a la carte multicomponent solid forms with tailored properties [8,9]. For instance, cocrystals with improved solubility [10,11], bioavailability [12], tabletability [13] or chemical stability [14] have been designed based on the toolbox provided by the so-called Crystal Engineering field of study [15].

**Figure 1:**
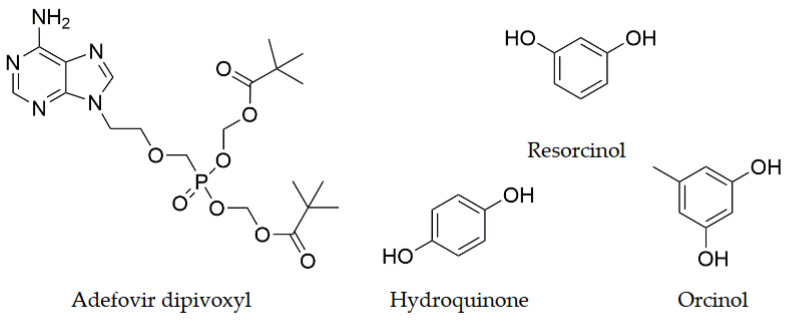
Molecular structures of Adefovir Dipivoxyl, and the coformers resorcinol, hydroquinone and orcinol.

Adefovir Dipivoxyl (9-{2-[Bis(pivaloyloxymethoxy)phosphinylmethoxy]ethyl}adenine) (AD, Figure 1) is a nucleotide reverse transcriptase inhibitor with antiviral activity against both HIV and HBV2, and it is effective against several other human viruses such as the hepatitis B virus [16,17]. This drug shows a diverse solid-forms landscape, including polymorphs [18], solvates [19], salts and cocrystals [20], which was revisited by some of us [21]. The drug shows a low solubility [17], a relatively low oral bioavailability [17,22] and chemical stability [23]. It has been more recently the subject of several solid-forms screens which have produced new cocrystals with paracetamol, saccharin and nicotinamide [24,25], suberic and succinic acid [26], gallic acid [27] and stearic acid [28]. Some of these cocrystals show improved physicochemical properties in terms of stability, solubility, dissolution or permeability.

With the aim to improve the oral bioavailability of Adefovir Dipivoxyl, we have searched for new cocrystals of AD guided by a Virtual Cocrystal Screen tool for the selection of potentially suitable coformers. After the synthesis and characterization of the new cocrystals, we have conducted a complete study of the dissolution of the new solid forms at relevant pH values of the gastrointestinal tract (GIT). Moreover, in order to better mimic the bioavailability along the GIT, we have performed dynamic dissolution studies adding an organic phase to simulate permeation into biological membranes while the pH changes from pH 1.5 (stomach) to pH 7.4 (physiological pH). 

## 2. Materials and Methods

### 2.1. Materials

Adefovir Dipivoxyl (AD) used in this study corresponds to its anhydrous form according to the comparison between the diffractogram of its bulk powder and the simulated diffractogram from the cif file Cambridge Crystallographic Data Centre (CCDC) refcode: TOYSUX, see Figure S1 for further details. AD (>99%) (provided by Urquima S. A., Palau-Solità i Plegamans, Spain). Quercetin (≥95.5%), Phloroglucinol (>99%), Resorcinol (>99%), Orcinol (≥95.5%) and toluene (>99.5%) were purchased from Sigma Aldrich (Darmastadt, Germany). Moreover, 4,4-Biphenol was purchased from ACROS (Thermo Fisher Scientific) (Geel, Belgium), Hydroquinone (>99%) was purchased from Panreac (ITW Reagents) (Castellar del Vallès, Barcelona, Spain) and Ethyl methyl ketone Scharlau (Scharlab) (Sentmenat, Barcelona, Spain).

For the dissolution experiments the following reagents were used: sodium acetate (>99.5%), potassium dihydrogen phosphate (>99.5%), 0.5 M potassium hydroxide Titrisol^®^ and 0.5 M hydrochloric acid Titrisol^®^, from Merck. Potassium chloride (>99%) and 1-decanol (>98%) were from Sigma-Aldrich (Burlington, MA, USA).

Water was purified by a Milli-Q plus system from Millipore (Bedford, MA, USA), with a resistivity of 18.2 MΩ cm.

### 2.2. Virtual Cocrystal Screening

Each compound was drawn in an extended conformation, and energy was minimized using the molecular mechanics methods implemented in TorchLite [29]. Gaussian 09 was used to optimize the geometry and calculate the Molecular Electrostatic Potential Surface (MEPS) on the 0.002 Bohr Å^−3^ electron density isosurface using Density Functional Theory (DFT) and a B3LYP/6-31G* basis set [30]. The MEPS was converted into Surface Site Interaction Points (SSIPs) using in-house software [31]. A total of 6 phenolic coformers with a probability of cocrystallization higher than 96% were chosen (see Table 1 for further details).

### 2.3. Cocrystal Screening

Qualitative solubilities of AD and each coformer in several organic solvents (24) were determined in order to design a set of experimental conditions for the cocrystal screening: liquid-assisted grinding (LAG) [32], reaction crystallization (RC) [33] and solvent-mediated transformation (SMT) experiments [34]. Evidences of new crystal forms have been observed through drop grinding (using a Retsch MM 2000 mill) with one drop of toluene. Evidences of new solid forms were deduced by comparing the X-ray Powder Diffraction (XRPD) patterns of all the known forms of AD and the coformers against the resulting solids. These evidences have been confirmed through SMT at 25 °C (50 mg of the final mixture in 1:1 molar ratio), and several scale up batches of the new cocrystals (50–600 mg of the final mixture) have been produced in order to perform dissolution rate and solubility studies (see ESI, Table S1 for further details). 

### 2.4. Synthesis of the Cocrystals

Synthesis of the anhydrous AD–resorcinol cocrystal (Form I) bulk powder was conducted by crystallization and solvent-mediated transformation. In particular, AD (500 mg, 0.977 mmol) and resorcinol (110 mg, 0.999 mmol) were mixed and dissolved in toluene (25 mL) at 80 °C for 30 min until total dissolution. Then, the solution was cooled down to 25 °C, and it was stirred for 24 h. The resulting suspension was filtered and dried under vacuum (2 h). Yield = 78%. Qualitative solubility of the AD–resorcinol cocrystal (Form I) in several organic solvents was also determined in order to design a set of experimental conditions for the preparation of single crystals suitable for X-ray analysis. Unfortunately, no high-quality single crystals were obtained through crystallizations at slow cooling rates, neither by means of slow solvent evaporations at 25 °C nor crystallization by antisolvent diffusion experiments (see ESI for further details). On the other hand, two new polymorphs of the AD–resorcinol cocrystal have been obtained through qualitative solubility experiments, and they also were characterized by means of Differential Scanning Calorimetry (DSC), XRPD and Nuclear Magnetic Resonance (^1^H-NMR). Unfortunately, the scale-up procedures for the two polymorphs could not be optimized (see ESI Section 2 and Section 3 for further details).

Synthesis of the anhydrous AD–orcinol cocrystal bulk powder was conducted by crystallization and solvent-mediated transformation. In particular, AD (500 mg, 0.977 mmol) and orcinol (124 mg, 0.999 mmol) were mixed and stirred in toluene (25 mL) at 80 °C for 30 min until total dissolution. Then, the solution was cooled down to 25 °C, and it was stirred for 24 h. The resulting suspension was filtered and dried under a vacuum (2 h). Yield = 91%.

Synthesis of the anhydrous AD–hydroquinone cocrystal bulk powder was conducted by crystallization and solvent-mediated transformation. In particular, AD (46 mg, 0.092 mmol) and hydroquinone (10 mg, 0.091 mmol) were mixed and stirred in ethyl methyl ketone (0.1 mL) at 40 °C for 30 min until total dissolution. Then, the solution was cooled down to 25 °C, and it was stirred for 24 h. The resulting suspension was filtered and dried under a vacuum (2 h). Yield = 80%.

### 2.5. Differential Scanning Calorimetry (DSC)

Differential scanning calorimetry measurements were carried out by means of a Mettler-Toledo (Columbus, OH, USA) DSC-822e calorimeter. Experimental conditions: aluminum crucibles of 40 μL, atmosphere of dry nitrogen with 50 mL/min flow rate and heating from 30 °C to 300 °C at a rate of 10 °C/min. The calorimeter was calibrated with indium of 99.99% purity (m.p.: 156.7 °C; ΔH: 28.37 J/g). 

### 2.6. Thermogravimetric Analysis (TGA) 

Thermogravimetric measurements were performed on a Mettler-Toledo (Columbus, OH, USA) TGA-851e thermobalance. Experimental conditions: alumina crucibles of 70 μL, atmosphere of dry nitrogen with 50 mL/min flow rate and heating from 30 °C to 300 °C at a rate of 10 °C/min. A blank curve has been previously performed by using the same methodology, and it has been subtracted.

### 2.7. X-ray Crystallographic Analysis

X-ray powder diffraction (XRPD) patterns of new cocrystals of AD were measured on a PANalytical (Malvern, UK) X’Pert PRO MPD (transmission configuration with Cu K*α* radiation, λ = 1.54187 Å) with a focalizing elliptic mirror and a PIXcel detector and a maximum detector’s active length of 3.347°. Transmission geometry configuration included a convergent beam with a focalizing mirror, a flat sample sandwiched between low absorbing films measuring from 1 to 40° in 2*θ*, a step size of 0.026° and a measuring time of 8 to 30 min at room temperature (25 °C). Capillary geometry has been used with AD–Resorcinol (Form I) placed in a glass capillary (Lindemman) 0.5 mm in diameter measuring from 2 to 70° in 2*θ*, with a step size of 0.013° and a total measuring time of 18 h. The powder diffractograms were indexed and the lattice parameters were refined by means of Le Bail fits by means of Dicvol04 [35], and the space group was determined from the systematic absences. *P*-1, *P*2 and *P*-1 space groups from AD cocrystals (resorcinol (form I), orcinol and hydroquinone, respectively) were deduced from the systematic absences and confirmed with the SGAid program of the *DAJUST* software [36]. 

### 2.8. Nuclear Magnetic Resonance (NMR)

Proton nuclear magnetic resonance (^1^H-NMR) spectra were measured on a Varian Mercury 400 (400 MHz) (Santa Clara, CA, USA) spectrometer. Chemical shifts for protons are reported in parts per million (ppm) downfield from tetramethylsilane (TMS) and referenced to the residual proton signal in the NMR solvent (dmso-*d*_6_: δ 2.50). Experimental conditions, delay: 1 s; pulse: 45°; scans: 32.

### 2.9. Intrinsic Dissolution Rate (IDR)

Three different sets of dissolution experiments were performed. In the first instance, dissolution measurements were carried out using an automatic titrator GLpKa from Sirius Instruments Ltd. (Forest Row, East Sussex, UK) with RefinementPro^TM^ software equipped with a Sirius KFP-1038 combined electrode, a D-PAS (10 mm of optical pass) and a photodiode array detector. The mentioned electrode, a stirrer, a temperature probe, capillary dispenser tubes and tubing for inert gas are included in the compact probe unit of the titrator.

The first set of studies was performed at different pH sectors (1.5, 4.0, 5.5 and 7.4) which are typically encountered in the gastrointestinal tract (GIT), and individual dissolution rates were determined at each pH sector. To perform these experiments, disks of 3mm diameter containing 6–10 mg of the drug or cocrystal were prepared by applying a constant pressure of 0.1 ton for 2 min with a manual hydraulic tablet press (Applied Measurements Specac Ltd, Orpington UK). The tablet was placed in a holder, and only one side of the tablet was exposed to the dissolution medium. The total exposed surface area was 0.07 cm^2^. A total of 1.5 mL of 0.125 M acetate–phosphate buffer at pH 1.6 was introduced into the vial without wetting the tablet surface. The instrument then automatically added 13.5 mL of 0.15 M KCl solution and adjusted the pH to the required value (see Table S2 for exact experimental details). Spectra collection started immediately after. Spectra were recorded every 30 s for 30 min. The medium was stirred at a constant rate. The solid state of the remaining disks was analyzed using XRPD.

A second set of experiments was performed with the same instrument. However, determinations were made in a full sequence of pHs (from 1.5 to 7.4) maintained for 30 min at each individual pH. To change the pH, KOH was added to the medium (Table S3).

The third set of measurements were performed using a Pion inForm automated titrator system from Pion (Pion-Inc., Billerica, MA, USA, www.pion-inc.com) with an incorporated UV-Vis spectrophotometer to acquire the spectrophotometric data. The optical system also consisted of a photodiode array detector with a deuterium lamp and included two fiber optic dip probes. The titrator module included a temperature controller (a Peltier device with in-situ thermocouple), pH electrode, an overhead stirrer and motorized dispensers for the automatic delivery of assay titrants and reagents via capillaries. The instrumentation was operated by inForm Control and Assay Designer software (version 1.6.0.0). The Pion inForm system was used for the biphasic dissolution studies. Samples were prepared as described previously, and studies were performed consecutively through the four different pH sectors, with the addition of a lipid layer (1-decanol) in the second sector to mimic the absorption in the GIT. The dissolution media consisted of 40 mL of aqueous solution, which was added automatically to an 80 mL dissolution vessel and brought to 25 °C (±0.1 °C). Once the media were adjusted to the starting pH and temperature, each sample was added automatically, and data collection was started from the point of sample introduction. The lipid layer (30 mL) was added in the second pH sector after the pH was adjusted to 4.0 (see Table S4 for exact experimental details). The two UV optic fiber probes each with a 10mm path length, were used on the inForm platform for quantitating the drug in the aqueous phase and in the lipid layer. Stirring of the solution was continuous at a rate of 100 rpm through the four sectors. Spectral and pH data were collected with a frequency of 30 s over a period of 2 h.

The calibration of both systems was carried out by standardizing 0.5 M potassium hydroxide base titrant with potassium hydrogen phthalate in triplicate. For GLpKa, 0.5 M hydrochloric acid titrant was standardized against the base titrant; for inForm, the hydrochloric acid titrant was standardized with tris(hydroxymethyl)aminomethane (TRIS). The pH electrode was calibrated daily using the Avdeef–Bucher four-parameter equation [37]. All data processing was performed with inForm Refine (version 1.6.0.0).

### 2.10. Extrapolated Dissolution Rate and Solubility Calculation

The concentration of AD in the solution at each time point was determined from the spectroscopic data by applying the Beer–Lambert law using the previously determined molar extinction coefficients. Spectra regions where the signal was saturated (A > 1.5) were discarded. The extrapolated dissolution rate was obtained through the fit of the first order Noyes–Whitney exponential equation to the data:

(1) $${\left[X\right]}_{t}=S\left(1-e^{-k_{d}\left(t-t_{0}\right)}\right)$$

In this equation, [*X*]*_t_* is the weight (g) of the compound in the solution at a given time (min), *S* is the extrapolated solubility (g) and *k_d_* is the dissolution rate constant (min^−1^). *t*_0_ (min) is a term allowing for a temporal offset. The dissolution rate is calculated through a refinement process in which *S*, *k_d_* and *t*_0_ optimal values are obtained by minimizing the root mean square deviation between the modelled concentrations and the measured ones. Then, the dissolution rate (g min^−1^) is obtained by the product *k_d_S* [38].

### 2.11. Dissolution Experiments Data Processing

AD concentration in the solution was determined from a previously measured sample Molar Extinction Coefficient (MEC) over a concentration range of 18–100 µM in aqueous and decanol conditions. Wavelength ranges of 260–310 nm and 244–285 nm were used for sample quantitation in the aqueous and decanol media, respectively. The UV contributions of coformers were also quantified using previously determined MECs over the same wavelength range. It should be noted that the coformers resorcinol, orcinol and hydroquinone absorbed practically at the same wavelengths as AD, although hydroquinone showed an additional UV signal from 288 nm. The MEC of AD and the three coformers, and the obtained pK_a_ values, are shown in Figure S21 and Table S5.

As seen in the MEC profiles, the spectra of resorcinol and orcinol overlapped with the one of AD in all the wavelength ranges. Thus, the data quantification in the GLpKa instrument was overestimated by about 10%. Moreover, the UV signal observed for AD–hydroquinone was saturated, and the UV data could not be processed from the first UV data point collected.

Contrary to GLpKa, which has a fixed probe with a 10 mm path length that cannot be exchangeable with different path length size tips, Pion inForm is provided with two UV probes, both with a 10 mm path length: a fixed path-length and an exchangeable path-length. These probes were used for the aqueous and the lipid media, respectively. In this way, the UV data collected for the three cocrystals could be quantified using the MECs of orcinol, resorcinol and hydroquinone. Moreover, in the inForm system a greater volume of solution is used (40 mL instead of 15 mL of water used for the GLpKa). Therefore, the signal was not saturated, and the UV spectra for the coformers and the adefovir could be distinguished between them.

## 3. Results and Discussion

### 3.1. Virtual Cocrystal Screen

**Figure 2:**
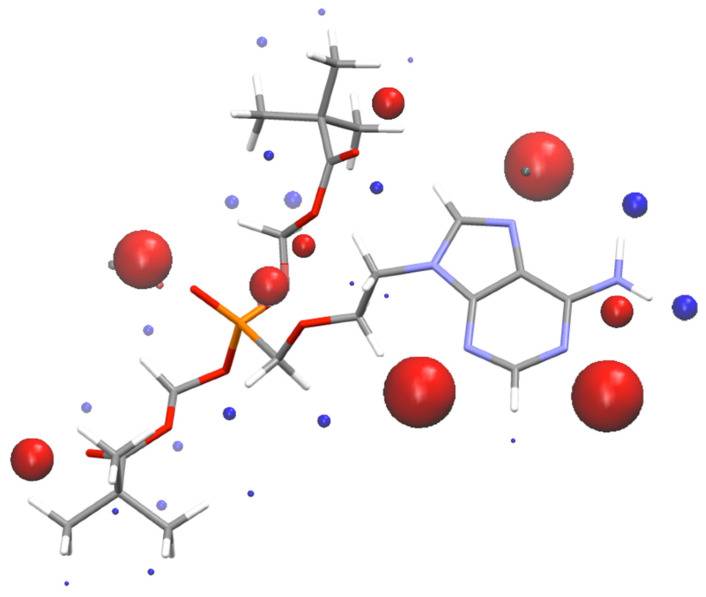
SSIPs calculated for Adefovir Dipivoxyl. Blue spheres correspond to H-bond donors, and red spheres to H-bond acceptors.

In order to guide the selection of a limited number of coformers for the experimental screen, we initially applied the virtual cocrystal screening methodology developed by Hunter [31]. This computational approach has been experimentally validated and used with success in several published studies [39,40,41,42,43,44]. This method is based on the calculation of the difference between the energy of the cocrystal and the sum of the energies of the pure API and the pure coformer, and the energy difference is used to produce a ranking of potential cofomers in a decreasing order of probability of cocrystal formation. The process requires the calculation of SSIPs from the ab initio MEPS of the isolated molecules in their most extended conformation. Then, the non-covalent interaction parameter (*ε_i_*) is determined for each SSIP, which is positive for an H-bond donor site (positive region on the MEPS) and negative for an H-bond acceptor site (negative region on the MEPS). The interaction energy between two SSIPs, *i* and *j*, is given by the product *ε_i_ε_j_*. The method is based on the assumption that SSIPs will be paired in a solid in order to maximize the total SSIP interaction energy, which allows for the evaluation of the interaction energies in the cocrystals and in the pure components without knowledge of their crystal structures. Thus, the energy of the three solid forms (drug, coformer and cocrystal) can be determined by pairing the most positive SSIP with the most negative SSIP, the next most positive SSIP with the next most negative, and so on, until all the potential interaction sites have been used. Thus, an estimation of the energy of each solid form, *E*, is provided by equation 2, and ∆*E* can be used to estimate the probability of cocrystal formation (Equation (3)).

(2) $$E=\sum \varepsilon_{i}\varepsilon_{j}$$

(3) $$\Delta E=-(E_{cc}-E_{1}-E_{2})$$

where *E*_1_, *E*_2_ and *E_cc_* are the total SSIP pairing energies of the pure API, coformer and its 1:1 cocrystal, respectively. The probability of cocrystallization is directly related to the ∆*E* value, which, by definition, is always positive. Following this methodology, we have screened all possible AD/coformer combinations using a coformer database which contains more than 2000 organic compounds (including 860 products from the generally regarded as safe list, GRAS). The coformers were ranked in order of decreasing ∆*E*. Since AD contains essentially strong hydrogen bond acceptors (Figure 2), and guided by previous successful experiences in crystal engineering with phenolic compounds [10,14,45,46,47,48], only 6 coformers belonging to this category of compounds were chosen from the most promising 50 coformers of the ranked list, (Table 1): quercetin, phloroglucinol, resorcinol, orcinol, 4,4-biphenol and hydroquinone. They have been highlighted in Table 1 in order to show their ranked position.

### 3.2. Characterization of the Cocrystals

Five multicomponent forms of AD have been obtained through a cocrystal screening with three out of the six coformers tested: three anhydrous forms with resorcinol (Form I, Form II and Form III), one anhydrous form with orcinol and one anhydrous form with hydroquinone. Each form has 1:1 stoichiometry (AD/coformer) deduced from ^1^H-NMR and TGA measurements and further characterized by means of DSC and XRPD.

**Figure 3:**
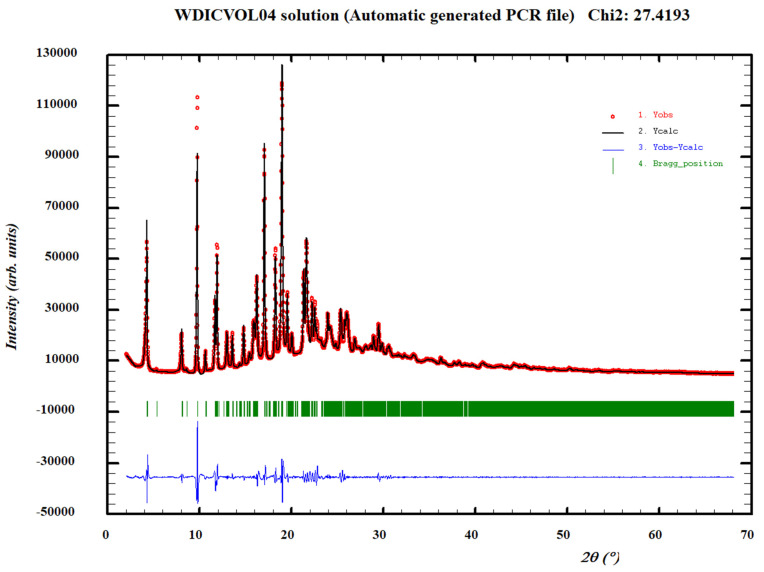
Pattern matching refinement of the AD–resorcinol cocrystal (Form I). Agreement factors: R_wp_: 5.06%; R_exp_: 0.97% (Chi-square: 27.42). Experimental XRPD profile (red marks), calculated XRPD profile (black solid line) and the difference between them (blue, line). Tick marks correspond to peak positions (|, in green).

The DSC analysis of the AD–resorcinol cocrystal (Form I) bulk powder shows an endothermic phenomenon at 96 °C, with an associated heat of 72.4 J/g (Figure S2, Supplementary Information). The TGA analysis does not show a weight loss before melting (Figure S3, Supplementary Information). The DSC analysis of the bulk powders of AD–Resorcinol cocrystal (Form II and III) show endothermic phenomena at 95 °C and 99 °C, with an associated heat of 81.1 J/g and 74.6 J/g, respectively (Figures S6 and S9, Supplementary Information). The TGA analysis for the two polymorphs could not be carried out due to a low amount of available powder, so they were assumed to be anhydrous because no traces of any solvent used in the synthesis were observed in the NMR analysis. On the other hand, although more than 100 different experimental conditions were performed aiming to obtain a single crystal, no good quality single crystals could be obtained for Single Crystal X-ray Diffraction (SCXRD) analysis. In parallel, high-quality data of the AD–resorcinol cocrystal bulk powder (Form I) were collected in capillary geometry to avoid preferential orientation problems in order to determine the crystal structure by means of direct space methodologies from powder X-ray diffraction data. Thus, the XRPD diffractogram was indexed at 298 K with the following proposed triclinic cell: a = 7.6735(5) Å, b = 18.2257 (12) Å, c = 22.4809 (6) Å, α = 112.784 (6)°, β = 94.311 (6)°, γ = 96.710 (4)° and V = 2853.9 (3) Å^3^ using Dicvol04, (Figures of Merit: *M*_20_ = 15.0, *F*_20_ = 41.4 (0.0069, 70)) with a number of impurities equal to zero. The cell volume is compatible with four molecules of AD and four molecules of resorcinol, Z = 4, (Z value according to estimated density (1.4 Mg m^−3^), Z’=2, (Figure 3). With two independent molecules of AD and two independent molecules of resorcinol in the asymmetric unit, we attempted to solve the crystal structure using the direct space strategy implemented in FOX [49] with the parallel tempering algorithm. Some constraints were introduced to FOX, considering phenol and 6-aminopurine rings as rigid groups. Several trials of 20 million runs were performed. Non-satisfactory structure solutions were obtained, probably caused by the high number of degrees of freedom, especially in the pivaloyloxymethyl protecting group [21].

**Figure 4:**
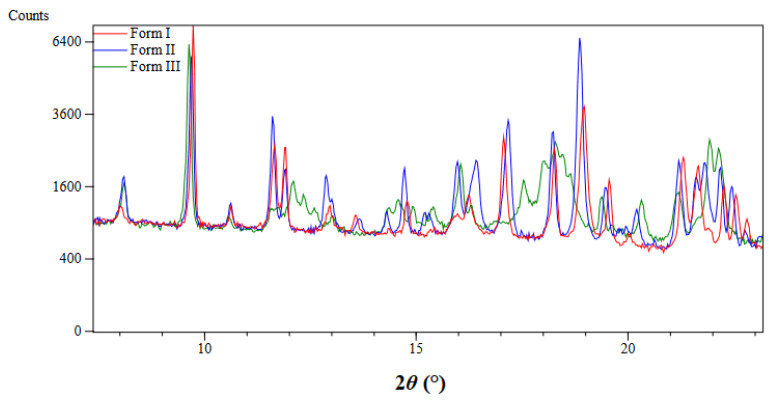
Comparative XRPD diffractograms of three polymorphs of the AD–resorcinol cocrystal: Form I (red), Form II (blue) and Form III (green). Enlargement from 7.5 to 23° in 2*θ* is represented for clarity.

The other two polymorphs of AD–resorcinol cocrystal (Forms II and III) have also been indexed in the triclinic system with good Le Bail fits (R_wp_: 6.95; R_exp_: 3.44 (Chi-square: 4.09) and R_wp_: 6.75; R_exp_: 3.49 (Chi-square: 3.73), respectively) (see Figures S7 and S10 for further details and Table 2 for comparison of cell parameters). Comparative XRPD diffractograms of the three polymorphs of the AD–resorcinol cocrystal are shown in Figure 4.

**Figure 5:**
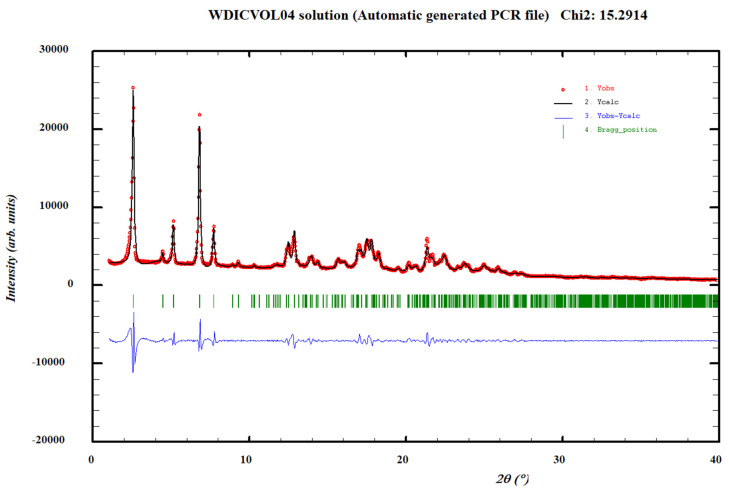
Pattern matching refinement of the AD–orcinol cocrystal. Agreement factors: R_wp_: 7.93%; R_exp_: 2.03% (Chi-square: 15.29). Experimental XRPD profile (red marks), calculated XRPD profile (black solid line) and the difference between them (blue, line). Tick marks correspond to peak positions (|, in green).

The DSC analysis of the AD–orcinol cocrystal bulk powder shows an endothermic phenomenon at 86 °C, with an associated heat of 67.0 J/g (Figure S13, Supplementary Information). The TGA analysis does not show a weight loss before melting (Figure S14, Supplementary Information). The bulk powder diffractogram of the anhydrous AD–orcinol cocrystal was indexed at 298 K with the following proposed monoclinic cell: a = 34.47 (2) Å, b = 19.81 (2) Å, c = 8.733 (7) Å, β = 93.63 (8)° and V = 5950 (8) Å^3^ by means of Dicvol04, (Figures of Merit: *M*_20_ = 10.2, *F*_20_ = 37.2 (0.0112, 48)) with a number of impurities equal to zero. The *P*2 space group was determined based on the assessment of systematic absences and confirmed with the SGAid program [36]. The cell volume is compatible with eight molecules of AD and eight molecules of orcinol, Z = 8, (according to estimated density of 1.4 Mg m^−3^), Z’ = 4, (Figure 5). As mentioned above, we did not attempt to solve the crystal structure using the direct space methodologies.

**Figure 6:**
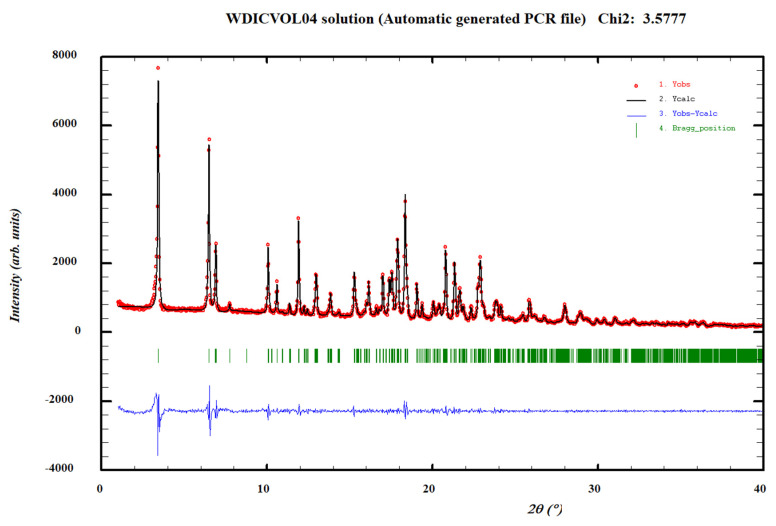
Pattern matching refinement of the AD–hydroquinone cocrystal. Agreement factors: R_wp_: 7.68%; R_exp_: 4.06% (Chi-square: 3.58). Experimental XRPD profile (red marks), calculated XRPD profile (black solid line) and the difference between them (blue, line). Tick marks correspond to peak positions (|, in green).

Finally, the DSC analysis of the AD–hydroquinone cocrystal bulk powder shows a first endothermic phenomenon at 72 °C, with an associated heat of 2.5 J/g, followed by a second endothermic phenomenon at 92 °C, with an associated heat of 73.7 J/g (Figure S17, Supplementary Information). The TGA analysis does not show a weight loss before melting (Figure S18, Supplementary Information). The bulk powder diffractogram of anhydrous AD–hydroquinone cocrystal was indexed at 298K with the following proposed triclinic cell: a = 39.826 (7) Å, b = 16.747 (4) Å, c = 8.392 (2) Å, α = 55.20 (1)°, β = 128.70 (1)°, γ = 135.58 (1)° and V = 2978 (1) Å^3^ by means of Dicvol04, (Figures of Merit: *M*_20_ = 22.2, *F*_20_ = 73.3 (0.0048, 57)) with a number of impurities equal to zero. The cell volume is compatible with four molecules of AD and four molecules of hydroquinone, Z = 4, (according to estimated density of 1.4 Mg m^−3^), Z’ = 2, (Figure 6). As mentioned above, we did not attempt to solve the crystal structure by means of direct space methodologies. Comparative crystallographic data from XRPD of AD cocrystals at 298 K are shown in Table 2.

Moreover, ^1^H-NMR analysis of all the AD cocrystals confirmed the 1:1 stoichiometric ratio based on the integration of resonance peaks for the AD and coformer in each cocrystal (see Figures S5, S8, S11, S16 and S20 Supplementary Information, for further characterization details).

### 3.3. Dissolution Rate Study

#### 3.3.1. Dissolution at Individual pH Sectors

**Figure 7:**
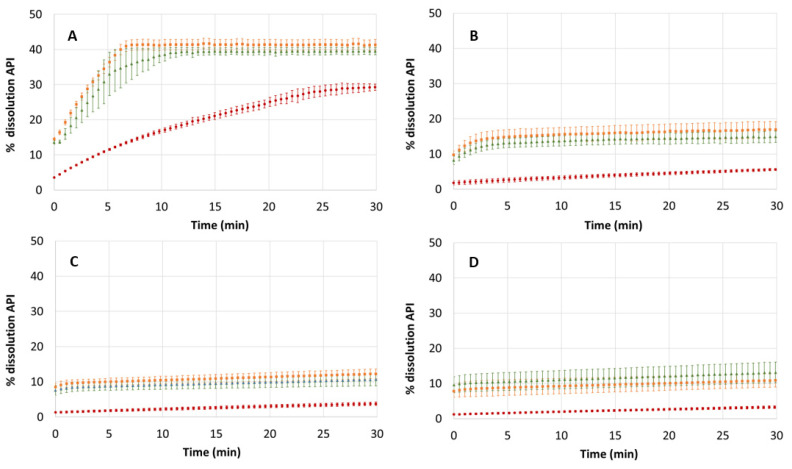
Dissolution profiles of AD (•), AD–Res cocrystal (Form I) (•) and AD–Orc cocrystal (•) at different pH values: pH 1.5 (**A**); pH 4.0 (**B**); pH 5.5 (**C**) and pH 7.4 (**D**).

In the first instance, the dissolution profiles of AD and the cocrystals of resorcinol (Form I) and orcinol were checked at four biorelevant pH values: 1.5, 4.0, 5.5 and 7.4. These are pH values encountered in different regions of the GIT and can provide important information about the behavior of the compound after oral intake. The data obtained with the cocrystal of hydroquinone was excluded from these determinations due to saturation problems explained in Section 2.11. Dissolution was monitored for 30 min, and the profiles are shown in Figure 7. The parameters obtained through the fit of Equation (1) to experimental data are shown in Table 3. In addition, Table 3 also shows the dissolution percentage reached after 30 min of dissolution and the solid form obtained after the dissolution experiments. The extrapolated dissolution rate (nmols min^−1^) was calculated when sink conditions were achieved, i.e., the experimental data points show a positive slope. When a negative or zero slope is obtained, the extrapolated dissolution rate cannot be calculated, as a negative slope denotes a precipitation event or that the compound is being partitioning to the lipid layer. At pH values different than 1.5, an initial rapid dissolution was observed for the cocrystals AD–resorcinol and AD–orcinol (in the range 1–3 min), followed by a decrease in the cocrystal dissolution until the end of the sector. This initial dissolution rate was not calculated because the number of collected data points in this range was not enough for an accurate determination. The solubility is calculated when saturated conditions are achieved. In this case, experimental data points must show slopes close to zero. Otherwise, an extrapolated solubility is obtained through Equation (1).

Figure 7 reveals that the dissolution rate of AD decreases as the pH increases. This can be also observed through the dissolution rate values in Table 3. The reason for this change is the ionization state of AD. At pH 1.5, AD is positively charged, a fact that favors the dissolution. However, at pH values higher than its pK_a_ (i.e. 4.0, 5.5, and 7.4), the percentage of neutral species is higher, and dissolution becomes slower. Compared to the dissolution of cocrystals, AD also shows a lower dissolution rate. At pH 1.5 (Figure 7A), the initial slope of the profiles is markedly higher for the cocrystals. According to Table 3, the dissolution of the cocrystals at this pH is 3.2-fold faster (resorcinol) and 2-fold faster (orcinol). In the case of cocrystals, the solution becomes saturated with AD after 5 min of dissolution, and for this reason, the % of API dissolved remains constant. These two plateaus indicate a solubility for AD around 300 μM. In contrast, the dissolution of the tablets that contain only AD is more gradual, and after 30 min of dissolution, the maximum solubility has not been reached yet. For the rest the of pH values (Figure 7B–D), the saturation concentration decreases (around 100 μM), particularly at pH 5.5 and 7.4, where AD exists only in its neutral form. Again, the cocrystals dissolve much faster, since the saturation concentration is reached after barely 5 min of dissolution.

The XRPD analysis of the tablet surface after the dissolution experiments confirms that there is some transformation of the solid form. The highest transformation occurs for AD tablets, where 20% of the initial AD (Form I) is transformed into the dihydrate. This transformation is reproducible, as deduced by the low error bars in the dissolution profiles. In the case of cocrystals, they mainly remain in its original solid form; the 5–10% of the free AD (Form I) observed in the analysis of the tablets could be the result of AD deposition during the experiments since the saturation concentration is reached very quickly, rather than a solid phase transformation in the surface of the tablet.

#### 3.3.2. Dissolution in Four pH Sectors Determination

**Figure 8:**
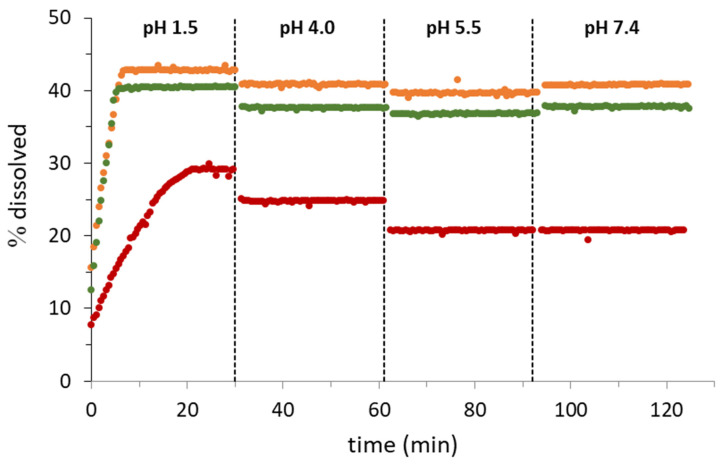
Dissolution profile of AD and two cocrystals in a four-pH sector test. AD (•), AD–Res cocrystal (Form I) (•) and AD–Orc cocrystal (•).

Compared to the single-pH experiments, the four-pH sectors determination provides dynamic information of the dissolution behavior of the compounds along the GIT. Now, the dissolution test starts at pH 1.5, and dissolution is monitored for 30 min. Then, the pH is raised to 4.0, 5.5, and finally, 7.4, monitoring dissolution for 30 min at each pH. Figure 8 shows the results obtained.

Dissolution at pH 1.5 follows the same pattern as in Figure 7A: fast dissolution for the cocrystals reaching 40% of API, dissolved until the saturation concentration is reached after 5 min, and a slowed down dissolution for the API, reaching a maximum of 30% of dissolution after 30 min. However, important differences are observed in the following pH sectors. Whereas in the individual dissolution tests at pH 4.0, 5.5 and 7.4, the amount of API dissolved decreased according to the lower solubility of AD at these pH values, in the dynamic test, the % of API dissolved is much higher. This is because whereas in the single-pH sector, determinations of the amount of AD in solution are limited by both the dissolution process and the solubility, in the 4-pH sector, determination of AD in the solution has already occurred when the change from pH 1.5 to pH 4.0 takes place. At this moment, precipitation of AD would be expected due to the lower solubility at pH 4.0. However, it remains stable in a supersaturated solution. The same occurs when the pH is changed from 4.0 to 5.5 and from 5.5 to 7.4. For the cocrystals the % of dissolution is practically the same as in pH 1.5 (40%), which demonstrates that AD is stable in supersaturated solutions, at least for the duration of these experiments. This fact may have a positive impact in the bioavailability through the GIT, partially overcoming the problems caused by the low solubility of this compound. For AD single solid form tablets, after a maximum dissolution at pH 1.5 (30%), the percentage of dissolved AD slightly decreases when the pH is increased. Nonetheless, the percentages in solution (25% at pH 4.0 and 20% at pH 5.5 and 7.4) are far away from the percentages obtained in the single-sector experiments (5.7, 3.8 and 3.3%, respectively) which points out that supersaturation also occurs for the single solid form during the dynamic process.

#### 3.3.3. Dissolution in a Biphasic System

**Figure 9:**
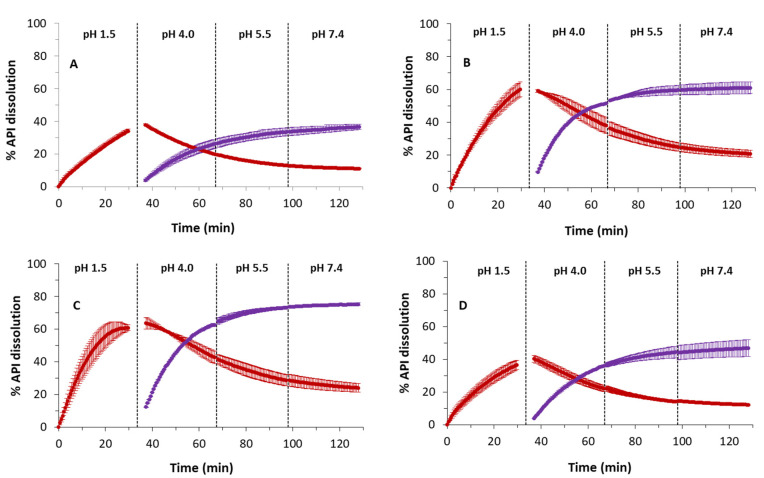
Dissolution profile of AD (**A**) and the AD–Resorcinol (Form I) (**B**), AD–Orcinol (**C**), and AD–Hydroquinone (**D**) cocrystals in a four-pH sectors biphasic test. (•) shows the AD percentage dissolved in the aqueous phase, and (•) the AD percentage dissolved in the lipidic phase.

In order to study dissolution under conditions that mimic in vivo conditions more closely than other systems, the final set of experiments was carried out in a biphasic dissolution system [50]. The test consists again in a four-pH sectors experiment, but in this case, 1-decanol was added in the second sector (pH 4.0) to simulate the contact with lipidic membranes. Then, the API concentration was monitored simultaneously in both phases through two different optic fiber probes. Figure 9 shows the obtained profiles.

Although dissolution profiles in the first pH sector seem similar to the ones in Figure 8, there are some aspects that should be noted. Firstly, dissolution of the hydroquinone cocrystal, which could not be evaluated in previous experiments, shows an inferior slope compared to the cocrystals with resorcinol and orcinol, revealing that this cocrystal does not improve the dissolution of AD as efficiently as the other two. The second aspect to remark upon is the higher percentage of dissolution reached in these determinations. This is caused by the higher amount of aqueous phase needed with this instrument. As a result, more AD from the tablet can be dissolved without reaching the maximum solubility.

The main differences appear once the 1-decanol phase is added, just after adjusting the pH to 4.0. The addition of the lipid layer mimics the flow of the compound from the stomach to the intestine, and the concentration of AD in the aqueous phase starts to decrease because of the simultaneous partition into the organic phase. This partition is more favorable when the pH is higher than the pK_a_ of AD (3.78 ± 0.05), where AD exists in its neutral form. In fact, the maximum concentration of AD in the organic solution is reached at pH 7.4 in all instances. However, the rate at which AD permeates to the organic phase is not equal for all the compounds. The dissolution at pH 4.0 shows that the cocrystals with resorcinol and orcinol show a rapid transfer of AD towards the organic layer, whereas this transfer is slightly slower for cocrystals with hydroquinone, and even slower for AD itself. This behavior is indicative that AD can permeate easily through the biological membranes administered in the form of a cocrystal, especially when resorcinol and orcinol are used as coformers. Another important factor to be noted is that this dynamic process displaces the equilibria, and this affects the initial dissolution of AD. The transfer of the compound to the organic layer favors the dissolution of the tablet, and higher concentrations of AD in solution are achieved. In fact, the percentage dissolved at the end of the first sector is lower than the sum of percentages (aqueous and organic phases) at the end of the last sector. This also occurs in vivo; thus, this type of experiment allows for a more realistic interpretation of how the different compounds will behave under real conditions and for selecting the best candidates to achieve the highest biodisponibility with the minimum dose.

## 4. Conclusions

New cocrystals of the antiviral drug Adefovir Dipivoxyl have been discovered by means of a combined virtual/experimental cocrystal screen, and their dissolution profiles have been further studied.

Dissolution of AD and their cocrystals at typical GIT pHs is markedly influenced by the ionization degree of the API. At pH 1.5 is where dissolution is faster, as AD is positively charged. However, the dissolution of the cocrystals of resorcinol and orcinol at this pH is 3.2- and 2-fold faster, respectively.

Dynamic dissolution determinations provide much more realistic information than the single-pH sector experiments. These determinations have shown that, on the one hand, sharp pH changes provoke AD supersaturated solutions. This is observed in the four-pH sectors experiments. After the first dissolution at pH 1.5, the solution remains supersaturated of AD in the three following pH sectors, the amount of AD dissolved being more or less constant along entire pH range. This fact may help in the biodisponibility of AD along the GIT, where pH changes accordingly. On the other hand, where an organic media that mimics biological membranes is added after the initial dissolution, AD partitions rapidly into the organic phase, especially at high pH values. However, the partition of AD from cocrystals of resorcinol and orcinol is faster than the partition of the AD from the hydroquinone cocrystal or the single AD solid form. Both factors, faster dissolution and faster partition into organic phases, point out that the oral bioavailability of AD should be higher when administered as resorcinol or orcinol cocrystal.

## Figures and Tables

**Table 1:** Coformers ranked based on Δ*E* and the probability of cocrystal formation with AD.

Rank	Coformer	Δ*E* (kJ mol^−^^1^)	P (%)
1	C-Methylcalix [4] resorcinarene	46.2	100
2	Sulfuric acid	33.5	99.99
3	1,2-Ethanedisulfonic acid	32.6	99.99
4	**Quercetin**	32.3	99.99
5	Gallic acid	32.1	99.99
6	**Phloroglucinol**	29.3	99.97
7	Resveratrol	28.5	99.96
8	3,5-Dihydroxybenzoic Acid	26.7	99.92
9	Tetracyanoethylene	26.1	99.90
10	2,3,5,6-tetrafluoro-7,7,8,8-tetracyanoquinodimethane	25.9	99.89
11	Phloretin	25.8	99.89
12	Diethylene glycol distearate	25.2	99.85
13	3,4-Dihydroxybenzoic Acid	25.2	99.85
14	**Resorcinol**	24.8	99.83
15	5-Nitroisophthalic Acid	24.3	99.79
16	4-Hexylresorcinol	24.0	99.77
17	D-erythro-Isocitric Acid	24.0	99.76
18	Sulfamic Acid	23.8	99.75
19	Nordihydroguaiaretic acid	23.4	99.70
20	Pyromellitic dianhydride	23.3	99.68
21	**Orcinol**	22.9	99.63
22	1,5-Naphthalenedisulfonic Acid	22.7	99.60
23	Eriodictyol	22.3	99.53
24	**4,4′-Biphenol**	21.7	99.41
25	Picric Acid	21.4	99.33
26	3,5-Dinitrobenzoic Acid	21.0	99.21
27	1,3,5-Benzenetricarboxylic Acid	20.9	99.18
28	Orotic acid	20.9	99.18
29	2-Methylsulfanylethyl acetate	20.8	99.15
30	2,4-Dihydroxybenzoic Acid	20.3	98.96
31	2,5-Dihydroxybenzoic Acid	20.3	98.95
32	Pyrogallol	19.9	98.79
33	4,4′-Cyclohexylidenebisphenol	19.7	98.70
34	**Hydroquinone**	19.6	98.61
35	Tartaric Acid	19.5	98.58
36	Citric Acid	19.3	98.48
37	1,2,4,5-Tetracyanobenzene	19.3	98.43
38	Imidazolidinyl urea	19.1	98.31
39	Chloranilic Acid	19.0	98.25
40	Camphoric Acid	18.6	97.93
41	Tetrahydroxy-1,4-quinone	18.4	97.81
42	(1R,2S)-1-Hydroxypropane-1,2,3-tricarboxylic Acid	18.2	97.64
43	Methyl gallate	18.1	97.56
44	Poly(vinyl acetate)	18.0	97.45
45	Cyanuric Acid	17.9	97.32
46	Menthyl valerate	17.8	97.25
47	Trifluoroacetic Acid	17.5	96.82
48	5-Sulfosalicylic Acid	17.4	96.71
49	Ethyl gallate	17.3	96.53
50	Propyl 3,4,5-trihydroxybenzoate	17.2	96.47

**Table 2:** Comparative crystallographic data from XRPD of AD cocrystals at 298 K.

Crystal Form	AD–Resorcinol			AD–Orcinol	AD–Hydroquinone
	Form I	Form II	Form III		
System	Triclinic	Triclinic	Triclinic	Monoclinic	Triclinic
Space group	*P*-1	*P*-1	*P*-1	*P*2	*P*-1
a (Å)	7.6735 (5)	29.157 (9)	29.77 (2)	34.47 (2)	39.826 (7)
b (Å)	18.2257 (12)	25.178 (9)	18.655 (9)	19.81 (2)	16.747 (4)
c (Å)	22.4809 (6)	7.694 (3)	10.021 (5)	8.733 (7)	8.392 (2)
α (°)	112.784 (6)	133.79 (2)	58.24 (5)	90	55.20 (1)
β (°)	94.311 (6)	130.27 (1)	134.67 (2)	93.63 (8)	128.70 (1)
γ (°)	96.710 (4)	47.82 (2)	101.55 (5)	90	135.58 (1)
Vol (Å^3^)	2853.9 (3)	2842 (2)	3230 (3)	5950 (8)	2978 (1)
Z	4	4	4	8	4
R_wp_ (%)	5.06	6.95	6.75	7.93	7.68

**Table 3:** Percentage of dissolution and dissolution rate (nmols min^−1^) of AD and the cocrystals at different GIT pH values. AD solubility (μM) and the solid forms observed in the tablet surface after the dissolution measurements are also provided. Standard deviation is shown in parenthesis (*n* =·3).

Compound	Starting Solid Form	pH	% Dissolved at t = 30 min	Dissolution Rate (nmols/min)	Solubility (μM)	R^2^	Final Solid Form
AD	Form I	1.5	29.0 (1.0)	315 (4)	325 (14)	>0.9882	Form I + AD–dihydrate (80%:20%)
		4.0	5.7 (0.4)	35 (4)	139 (19) *	>0.9998	
		5.5	3.8 (0.6)	21 (4)	128 (32) *	>0.9999	
		7.4	3.3 (0.4)	17 (2)	123 (25) *	>0.9998	
AD–Res cocrystal (Form I)	Cocrystal (Form I)	1.5	39.3 (1.3)	1015 (6)	304 (13)	>0.9850	Cocrystal (Form I) + AD–dihydrate (95%:5%)
		4.0	16.1 (2.0)	63 (2)	129 (22)	>0.8406	
		5.5	12.2 (2.0)	19 (1)	109 (33) *	>0.9982	
		7.4	10.9 (2.7)	15 (1)	105 (10) *	>0.9982	
AD–Orc cocrystal	Cocrystal	1.5	39.2 (1.1)	626 (40)	289 (9)	>0.9803	Cocrystal + AD–dihydrate (90%:10%)
		4.0	14.7 (1.6)	147 (47)	138 (25)	>0.8292	
		5.5	10.6 (2.5)	17 (1)	92 (3)	>0.9992	
		7.4	13.1 (3.6)	17 (3)	88 (7)	>0.9809	

* Extrapolated solubility (from Equation (1)).

## Data Availability

Data are provided in the supplementary material.

## Supplementary Materials

The following supporting information can be downloaded at: https://www.mdpi.com/article/10.3390/pharmaceutics14112310/s1, Figure S1: Comparative PXRD diffractograms of adefovir dipivoxil powder used as starting material and simulated from the cif files; Figure S2: DSC of AD–resorcinol cocrystal (Form I); Figure S3: TGA of AD–resorcinol cocrystal (Form I); Figure S4: Comparative PXRD diffractograms of adefovir dipivoxil powder used as starting material, resorcinol, and AD–resorcinol cocrystal (Form I); Figure S5: ^1^H-NMR (dmso-d_6_/delay: 1 s /pulse: 45°/scans: 32) of AD–resorcinol cocrystal (Form I) (1:1); Figure S6: DSC of AD–resorcinol cocrystal (Form II); Figure S7: XRPD of the AD–resorcinol cocrystal (Form II); Figure S8: ^1^H-NMR (dmso-d_6_/delay: 1 s /pulse: 45°/scans: 32) of AD–resorcinol cocrystal (Form II) (1:1); Figure S9: DSC of AD–resorcinol cocrystal (Form III); Figure S10: XRPD of the AD–resorcinol cocrystal (Form III); Figure S11: ^1^H-NMR (dmso-d_6_/delay: 1 s /pulse: 45°/scans: 32) of AD–resorcinol cocrystal (Form III) (1:1); Figure S12: Comparative XRPD diffractograms of the three polymorphs of AD–resorcinol cocrystal; Figure S13: DSC of AD–orcinol cocrystal; Figure S14: TGA of AD–orcinol cocrystal; Figure S15: Comparative PXRD diffractograms of adefovir dipivoxil powder used as starting material, orcinol, and AD–orcinol cocrystal; Figure S16: ^1^H-NMR (dmso-d_6_/delay: 1 s /pulse: 45°/scans: 32) of AD–orcinol cocrystal (1:1); Figure S17: DSC of AD–hydroquinone cocrystal; Figure S18: TGA of AD–hydroquinone cocrystal; Figure S19: Comparative PXRD diffractograms of adefovir dipivoxil powder used as starting material, hydroquinone, and AD–hydroquinone cocrystal; Figure S20: ^1^H-NMR (dmso-d_6_/delay: 1 s /pulse: 45°/scans: 32) of AD–hydroquinone cocrystal (1:1); Figure S21: MEC absorption profiles of the different species of Adefovir Dipivoxyl and the neutral species of the coformers; Table S1: Cocrystal screening of AD; Table S2: Experimental conditions for the single sector pH dissolution assays; Table S3: Experimental conditions for the four sector pH dissolution assay; Table S4: Experimental conditions for the four sector pH biphasic-dissolution assays; Table S5: pK_a_ values of the compounds under study at 25 °C and zero ionic strength.
