# Supplementary material for: Virtual Cocrystal Screening of Adefovir Dipivoxyl: Identification of New Solid Forms with Improved Dissolution and Permeation Profiles
Source: Pharmaceutics. 2022 Oct 27;14(11):2310. doi: 10.3390/pharmaceutics14112310 (PMC9692677; doi:10.3390/pharmaceutics14112310)
Supplement: Supplementary file 1 [file pharmaceutics-14-02310-s001.zip › pharmaceutics-1935014-supplementary.pdf]

Virtual Cocrystal Screening of Adefovir Dipivoxyl: Identification of New Solid Forms with Improved Dissolution and Permeation Profiles

Rafael Barbas [1], Hanan Fael [2], Samuel Lee [3], Rebeca Ruiz [3], Christopher A. Hunter [4], Elisabet Fuguet [2,5], Clara Ràfols [2,*] and Rafel Prohens [1,*]

[1] Unitat de Polimorfisme i Calorimetria, Centres Científics i Tecnològics, Universitat de Barcelona, Baldiri Reixac 10, 08028 Barcelona, Spain
[2] Departament d'Enginyeria Química i Química Analítica, Universitat de Barcelona, Martí i Franquès 1-11, 08028 Barcelona, Spain
[3] Pion Inc., Forest Row Business Park, Forest Row, East Sussex RH18 5DW, UK
[4] Yusuf Hamied Department of Chemistry, University of Cambridge, Cambridge CB2 1EW, UK
[5] Serra Húnter Programme, Generalitat de Catalunya, 08017 Barcelona, Spain
* Correspondence: crafols@ub.edu (C.R.); rafel@ccit.ub.edu (R.P.)

Electronic Supplementary Information

Table of contents:

1. Materials and methods……………………………………………….…………….2

2. Cocrystal screening experiments …………………………………………………4

3. Synthesis of new AD-resorcinol cocrystal polymorphs ……………………......5

4. Characterization of AD cocrystals …………………………………………......6

5. Experimental conditions for the dissolution assays...…………..………………25

6. Determination of the Molar Extinction Coefficients and acidity constants….28

1. Materials and methods

1.1 Materials

Adefovir dipivoxil (AD) used in this study corresponds to its anhydrous crystal form, Figure S1.

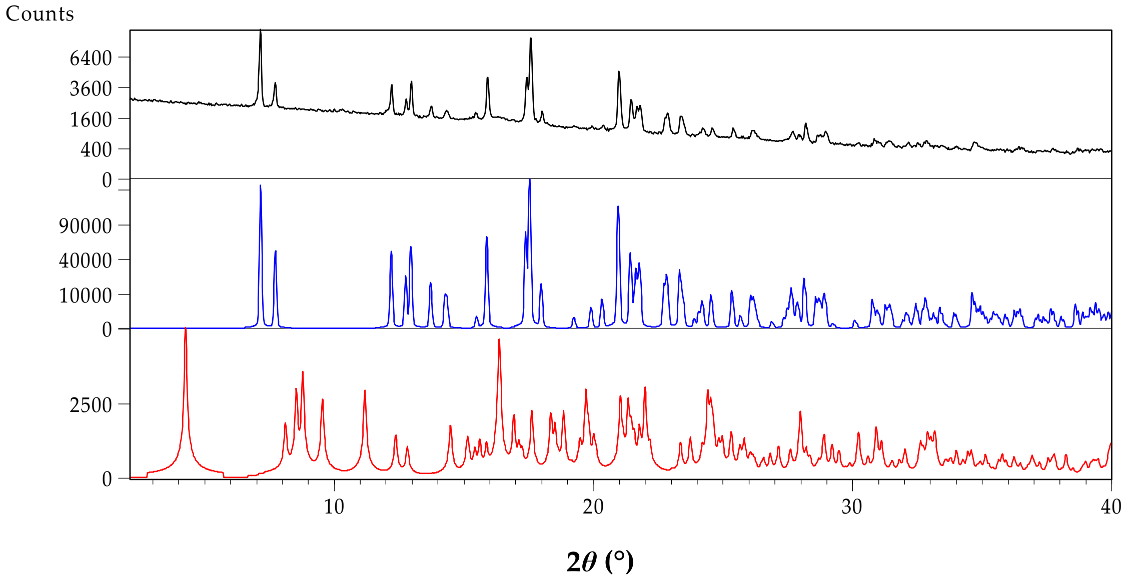

Figure S1. Comparative PXRD diffractograms of adefovir dipivoxil powder used as starting material (black) and simulated from the cif files: adefovir dipivoxil anhydrous (CCDC refcode: TOYSUX) (blue) and adefovir dipivoxil dihydrate (CCDC refcode: JEVQUX) (red)

1.2 Cocrystal Screening Methodologies

1.2.1 Liquid assisted grinding methodology at 25 °C

Screening through liquid-assisted grinding experiments (LAG) was conducted by grinding 20-50 mg of a 1:1 mixture of AD and each coformer together with one drop of different solvents using a Retsch MM 2000 grinding mill. The samples were placed in 2 mL volume stainless steel jars, along with two stainless tungsten grinding balls of 3 mm diameter. Grinding was performed for 15-30 minutes, with a mill frequency of 30 Hz.

1.2.2. Reaction crystallization methodology

Reaction crystallization experiments (RC) was conducted by preparing a saturated solution of resorcinol in water in a sealed vial under stirring. A small quantity of AD was added until it did not dissolve anymore. A collide was obtained after 24 hours and the resulting solution was not filtered.

1.2.3. Solvent mediated transformation methodology at 25 °C

Screening through solvent mediated transformations (SMT) were conducted by preparing suspensions of AD and coformer in different molar ratios (20-30 mg of the final mixture) in suitable solvents. The sealed vials were stirred for different times and the resulting solids were filtered and analyzed by XRPD.

1.2.4. Crystallizations at slow cooling rate

Solutions of AD and coformer mixtures in 1:1 molar ratio (10-20 mg of the final mixture) were prepared in different solvents at 25 °C and heated up (to 50 °C or 60 °C) in a heating stainless steel block. The heater was switched off and the solutions were allowed to slowly cool down to 25 °C inside the heating block. The samples which did not crystallize were tightly sealed and kept at 4-8 °C or 25 °C until precipitation was observed.

1.2.5. Slow evaporations at 25 °C

Solutions of AD and coformer mixtures in 1:1 molar ratio (10-20 mg of the final mixture) were dissolved in different solvents. The solutions were kept in opened vials at 25 °C while the solvent slowly evaporates until crystallization of a solid occurs.

1.2.6. Crystallizations by antisolvent diffusion

Solutions of AD and coformer mixtures in 1:1 molar ratio (10-20 mg of the final mixture) were dissolved in different solvents at 25 °C and an antisolvent was let to diffuse into the solution inside a bigger tube closed to the air.

1.2.7. Preparation of the new solid forms: scale up batches

Experimental procedures for the preparation of resorcinol, orcinol and hydroquinone cocrystals have been optimized and several scale up batches were prepared for dissolution rate and pharmacokinetics studies. The main procedures of the anhydrous AD cocrystals bulk powders were conducted by crystallizations and solvent-mediated transformations.

2.- Cocrystal screening experiments

Table S1. Cocrystal screening of AD.

Methodology	Coformers	Nº Experiments	Nº Solids	Positive results [a]	Coformers	Form obtained (according to XRPD)
Solubility Study	-	24	-	-	-	-
Liquid assisted grinding at 25 °C	6	44	43	1	3	AD:RE I, AD:OR and AD:HY
Reaction crystallization	1	1	-	-	-	-
Solvent mediated transformation at 25 °C	3	3	3	2	3	AD:RE I, AD:OR and AD:HY
Crystallizations at slow cooling rate	2	3	3	1 / 2	1	AD:HY
Slow evaporations at 25 °C	2	52	39	2	2	AD:RE and AD:HY
Crystallization by antisolvent diffusion	2	61	48	-	-	-
Preparation of the solid forms: scale up batches	3	9	8	2	3	AD:RE I, AD:OR and AD:HY

[a] (1) positive: AD + coformer + new peaks observed in XRPD, (2) positive: cocrystal

3. Synthesis of new AD-resorcinol cocrystal polymorphs

3.1 AD- resorcinol cocrystal (1:1)

3.1.1 AD-resorcinol cocrystal (Form I): qualitative solubility determination

In order to obtain a single crystal of AD–resorcinol cocrystal (Form I), qualitative solubility was determined. Thus, the AD–resorcinol cocrystal (Form I) (10 mg, 0.016 mmol) was dissolved in 29 solvents in a temperature range of 25-60 °C. AD-resorcinol cocrystal (Form I) is soluble at 25 ºC in the following solvents: ethylene glycol (0.1 mL), DMF (0.1 mL), DMSO (0.1 mL), diethyl ether (0.1 mL), dichloromethane (0.1 mL), chloroform (0.1 mL), acetic acid (0.1 mL) and NH_3 (32 %) in water (0.1 mL). At 50 ºC it is soluble in ethanol (0.5 mL), IPA (0.6 mL), *tert*-butanol (0.6 mL) and butanol (0.6 mL). It is partially soluble at 50 ºC in methanol (1.5 mL), ACN (1.0 mL), AcOEt (1.0 mL), MEK (1.0 mL), acetone (1.0 mL), MiBK (1.0 mL), THF (1.0 mL), dioxane (1.0 mL), methyl *tert*-butyl ether (2.0 mL) and dimethyl ethylene glycol (1.0 mL). At 60 ºC it is soluble in toluene (1.5 mL) and xylene (1.5 mL). it is insoluble in water (1.0 mL), pentane (1.0 mL), heptane (1.0 mL), cyclohexane (1.0 mL) and diisopropyl ether (1.0 mL).

Synthesis of the new AD-resorcinol cocrystal polymorphs (Form II and Form III) were conducted by crystallization methodologies after qualitative solubility determination of AD–resorcinol cocrystal (Form I). Details of synthesis are as follows:

3.1.2 AD- resorcinol cocrystal (Form II)

AD-resorcinol cocrystal (Form II) was obtained by recrystallization of AD-resorcinol cocrystal (Form I) ((10 mg, 0.016 mmol) in xylene after 24 hours at 25 °C.

3.1.3 AD-resorcinol cocrystal (Form III)

AD-resorcinol cocrystal (Form III) was obtained by slow evaporation of AD-resorcinol cocrystal (10 mg, 0.016 mmol) solutions in diethyl ether, dichloromethane, AcOEt and chloroform after 24 hours, 24 hours, 8 days and 8 days at 25 °C, respectively.

4.- Characterization of AD cocrystals

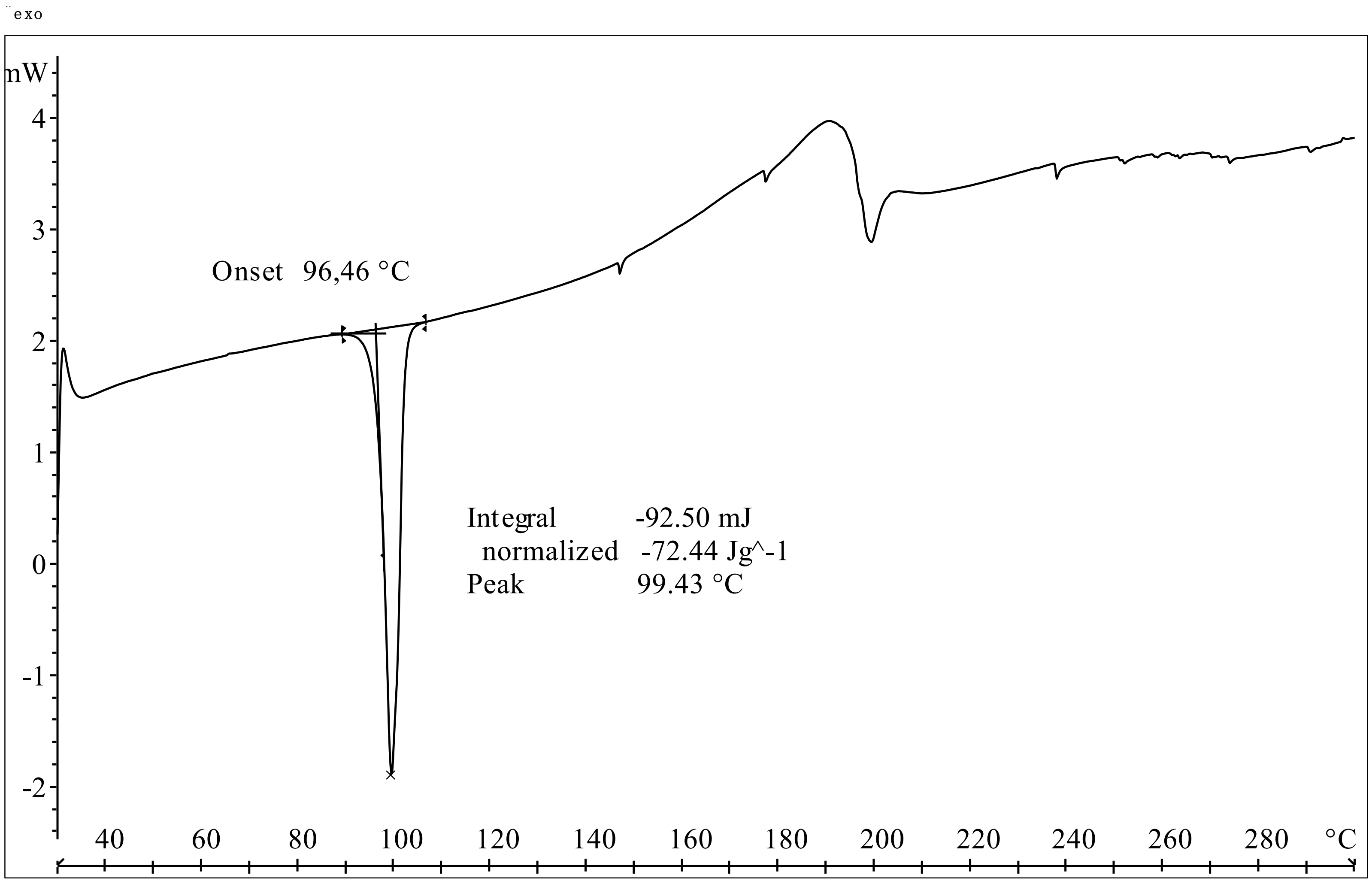

Figure S2. DSC of AD–resorcinol cocrystal (Form I).

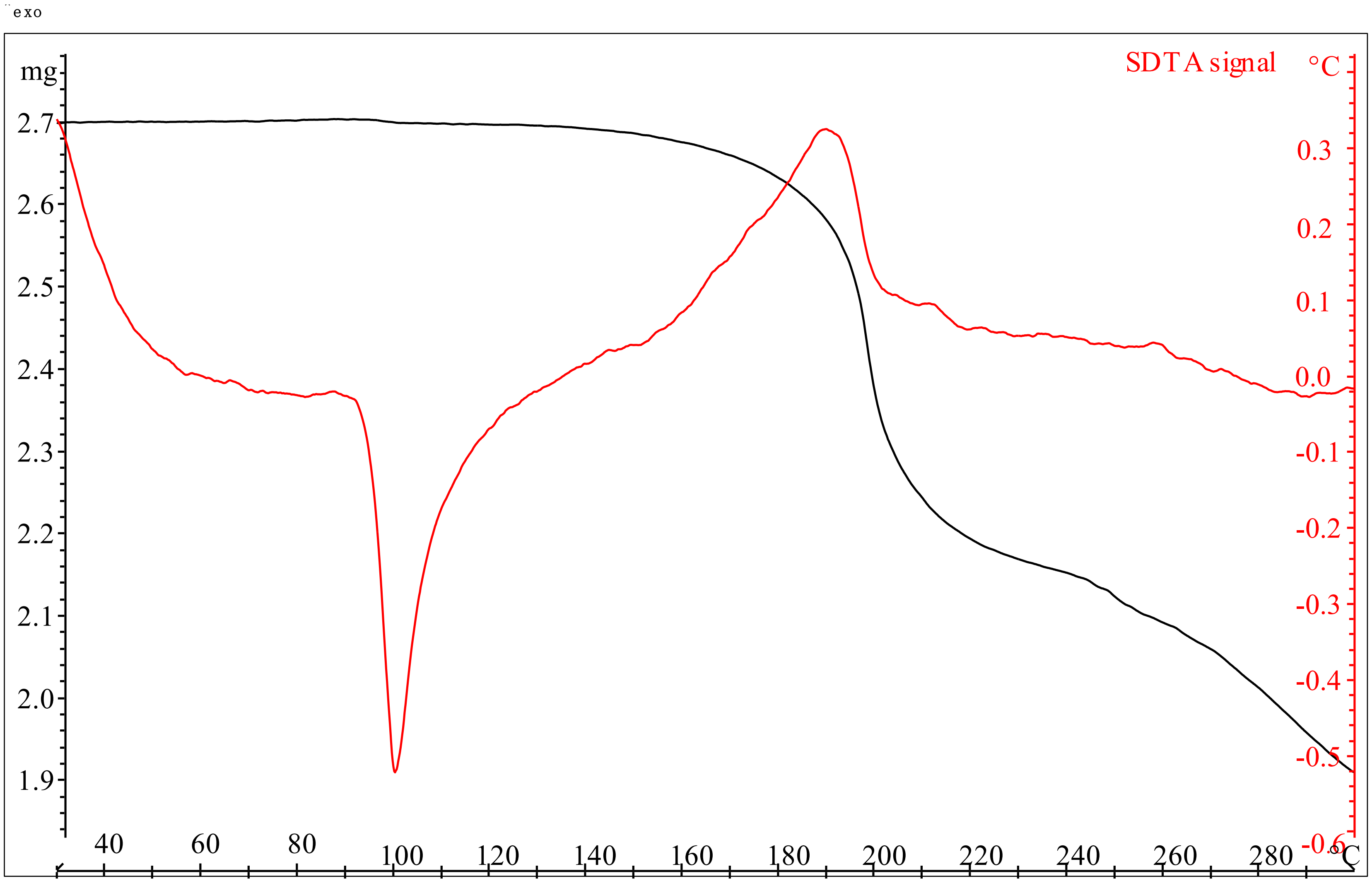

Figure S3. TGA of AD–resorcinol cocrystal (Form I): the TGA analysis does not show a weight loss before melting.

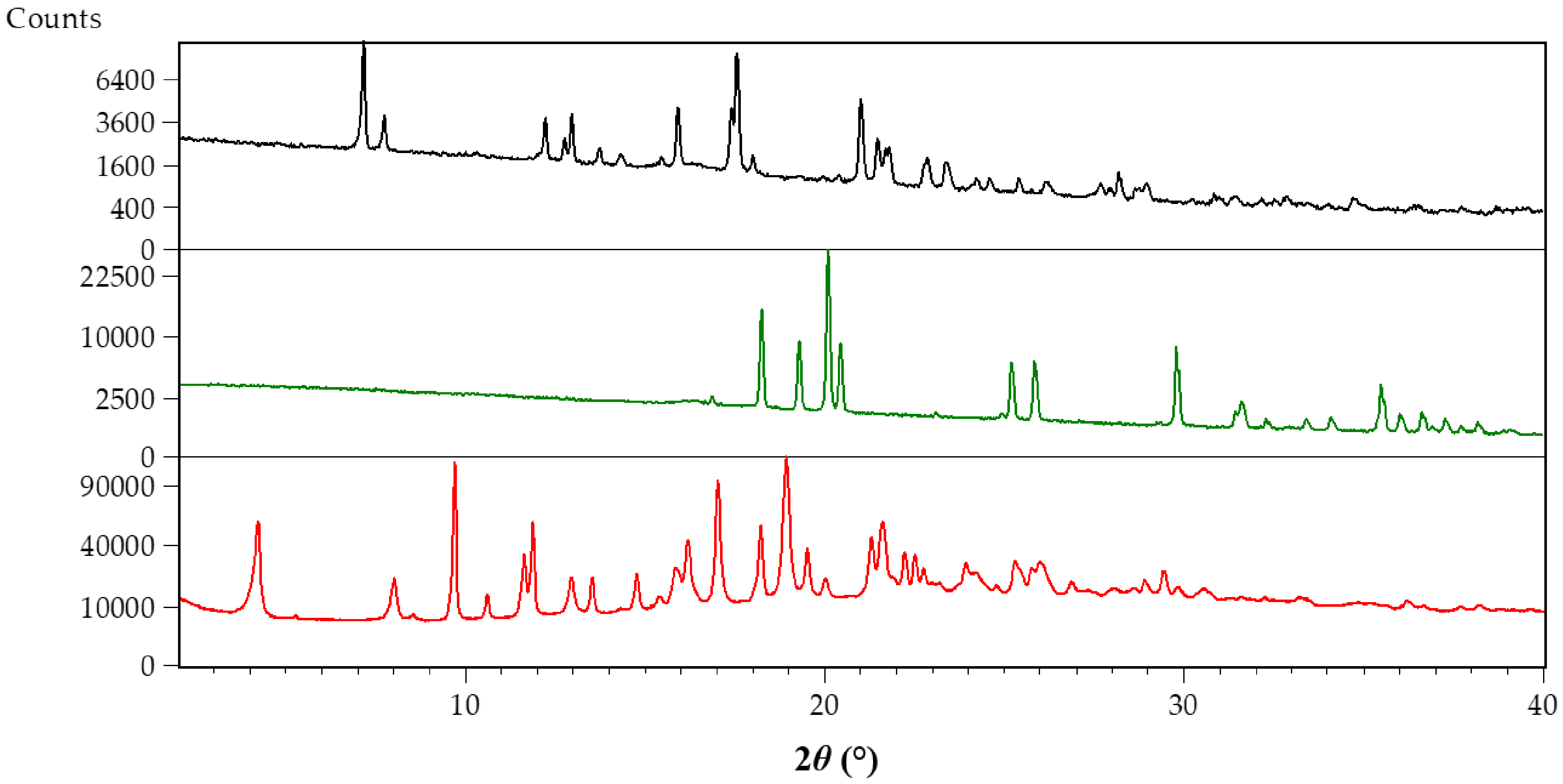

Figure S4. Comparative PXRD diffractograms of adefovir dipivoxil powder used as starting material (black), resorcinol (green) and AD-resorcinol cocrystal (Form I) (red).

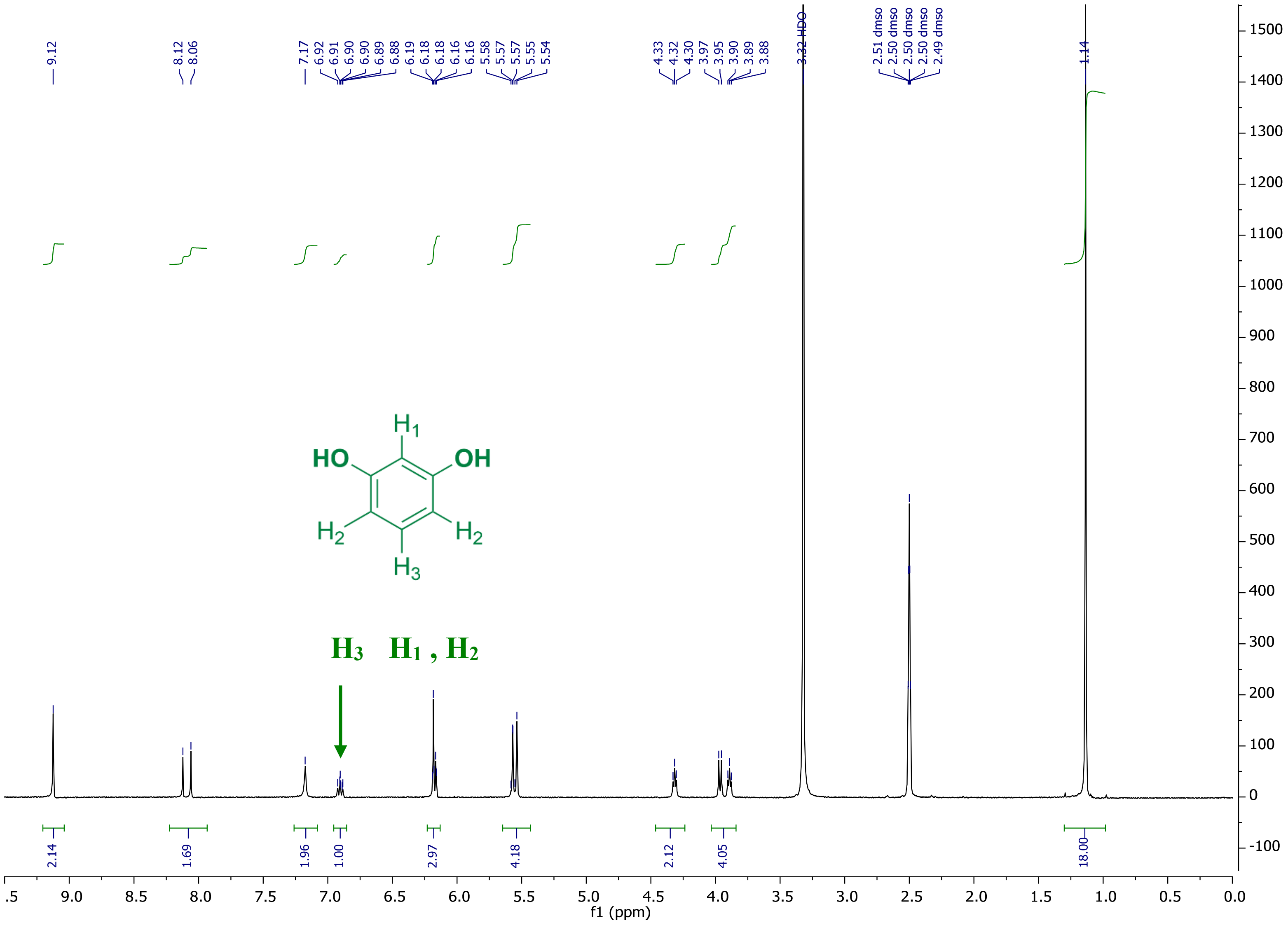

Figure S5. ^{1}H-NMR (dmso-d_6/delay: 1 second /pulse: 45°/scans: 32) of AD–resorcinol cocrystal (Form I) (1:1).

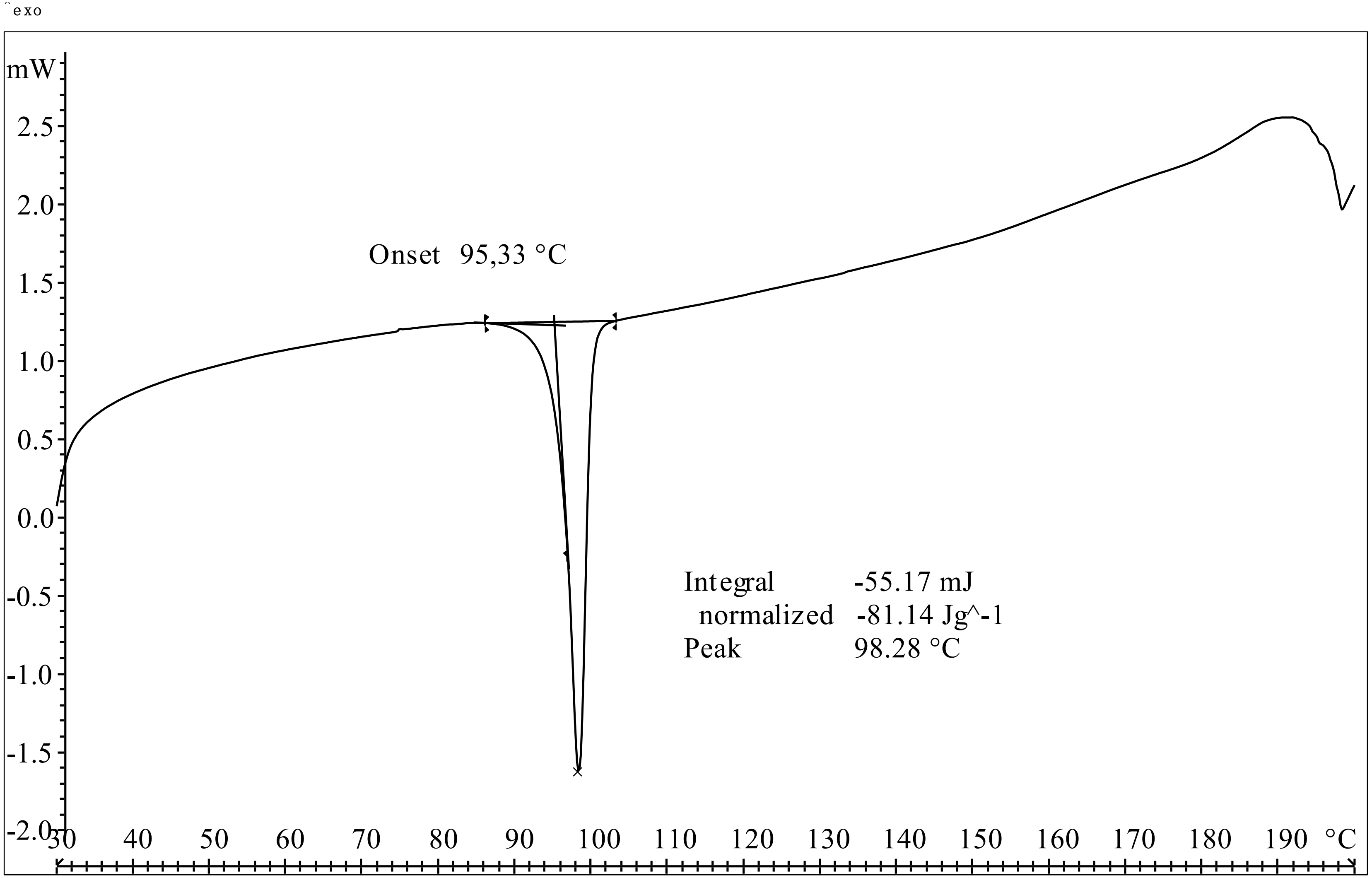

Figure S6. DSC of AD–resorcinol cocrystal (Form II).

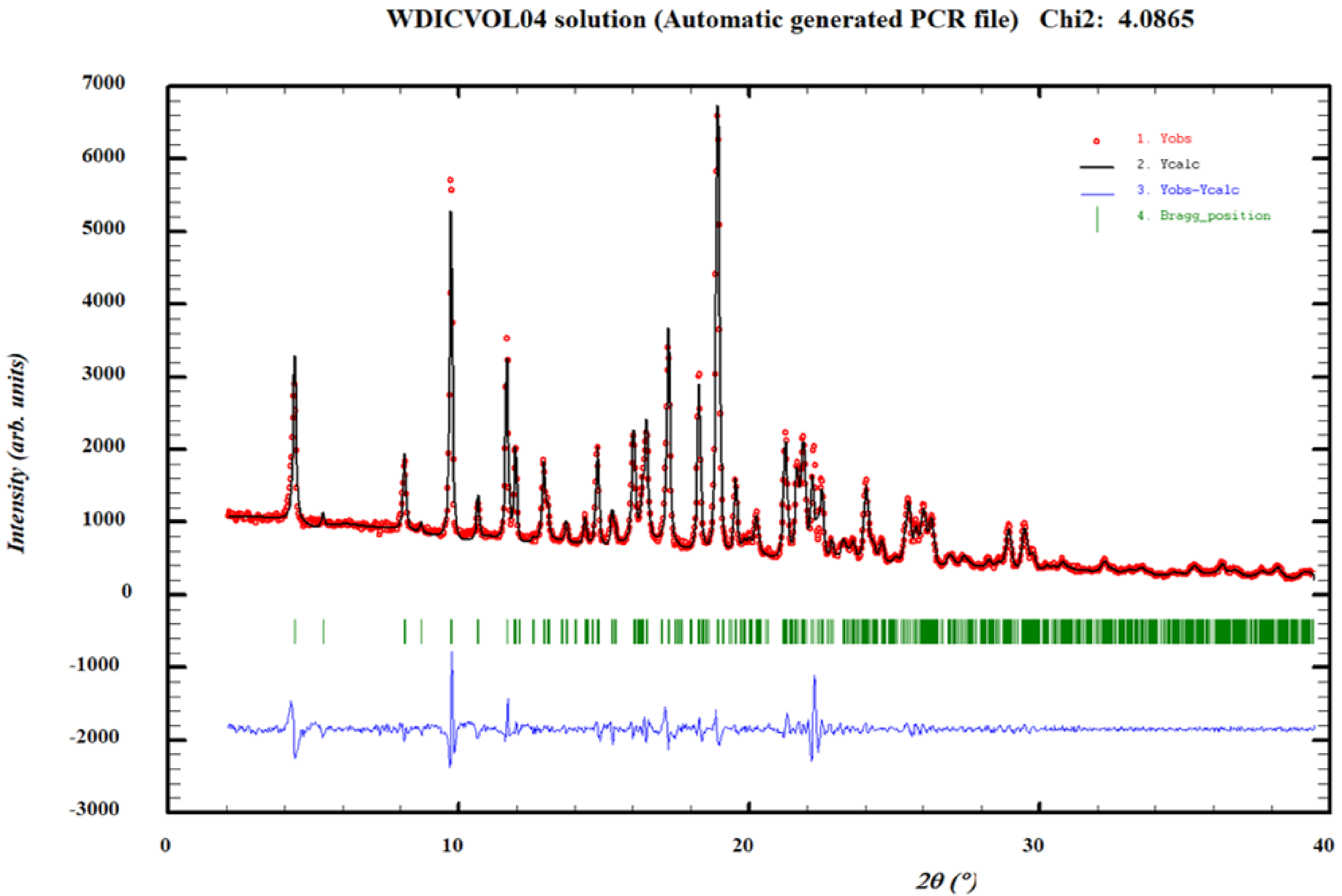

Figure S7. The XRPD of the AD-resorcinol cocrystal (Form II) has been indexed at 298 K with the following proposed triclinic cell: a=29.157(9) Å, b=25.178(9) Å, c=7.694(3) Å, α= 133.79(2)°, β= 130.27(1)°, γ= 47.82(2)° and V=2842(2) Å^3 (Figures of Merit: M_{20}= 17.1, F_{20}= 51.3 (0.0054, 72)), with number of impurities equal to zero. The cell volume is compatible with 4 molecules of adefovir dipivoxil and 4 molecules of resorcinol. (R_{wp}: 6.95; R_{exp}: 3.44), Z=4, (Z value according to estimated density of 1.4 Mg m^{-3}), Z'=2.

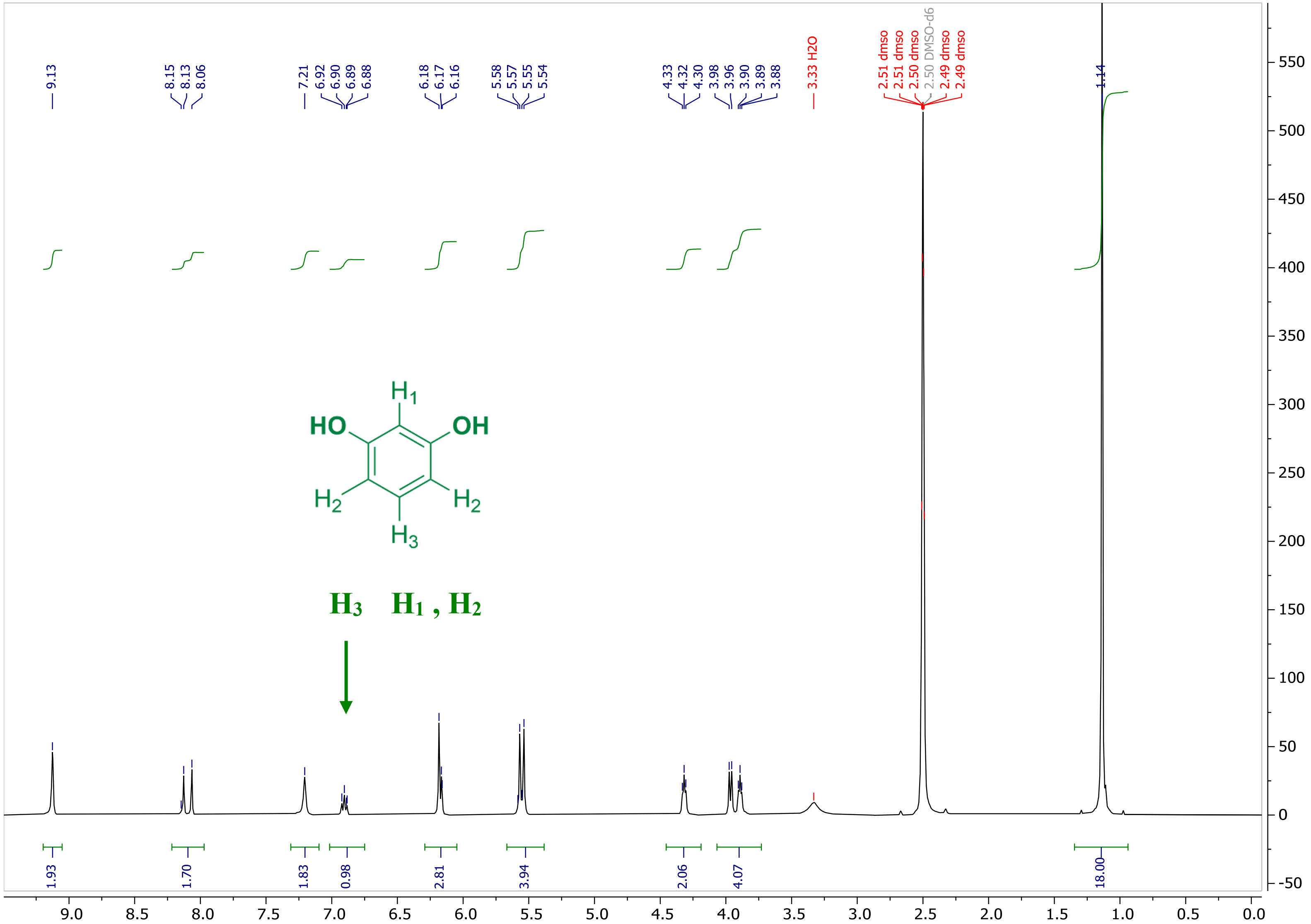

Figure S8. ^{1}H-NMR (dmso-d_6/delay: 1 second /pulse: 45°/scans: 32) of AD–resorcinol cocrystal (Form II) (1:1).

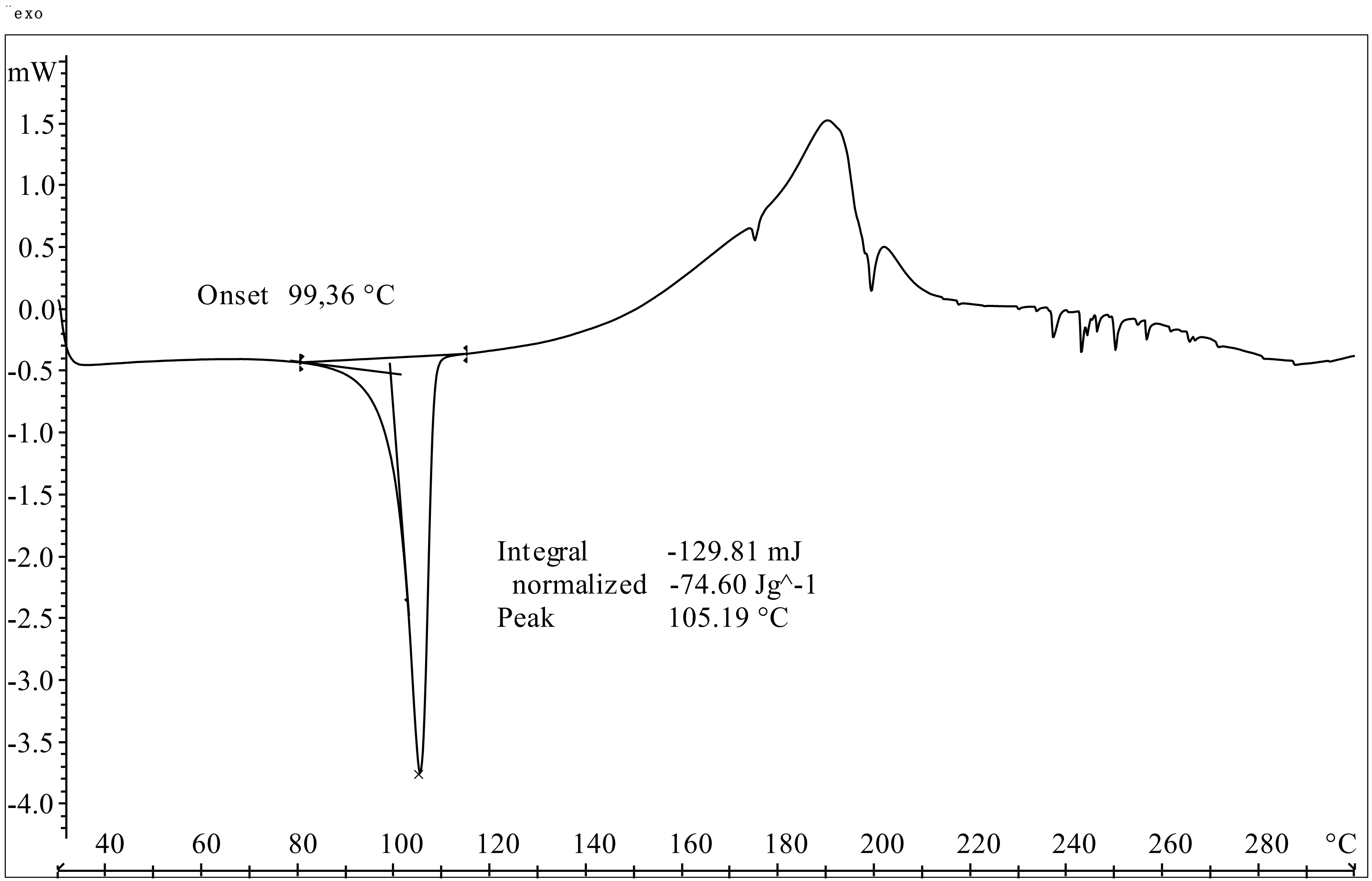

Figure S9. DSC of AD–resorcinol cocrystal (Form III).

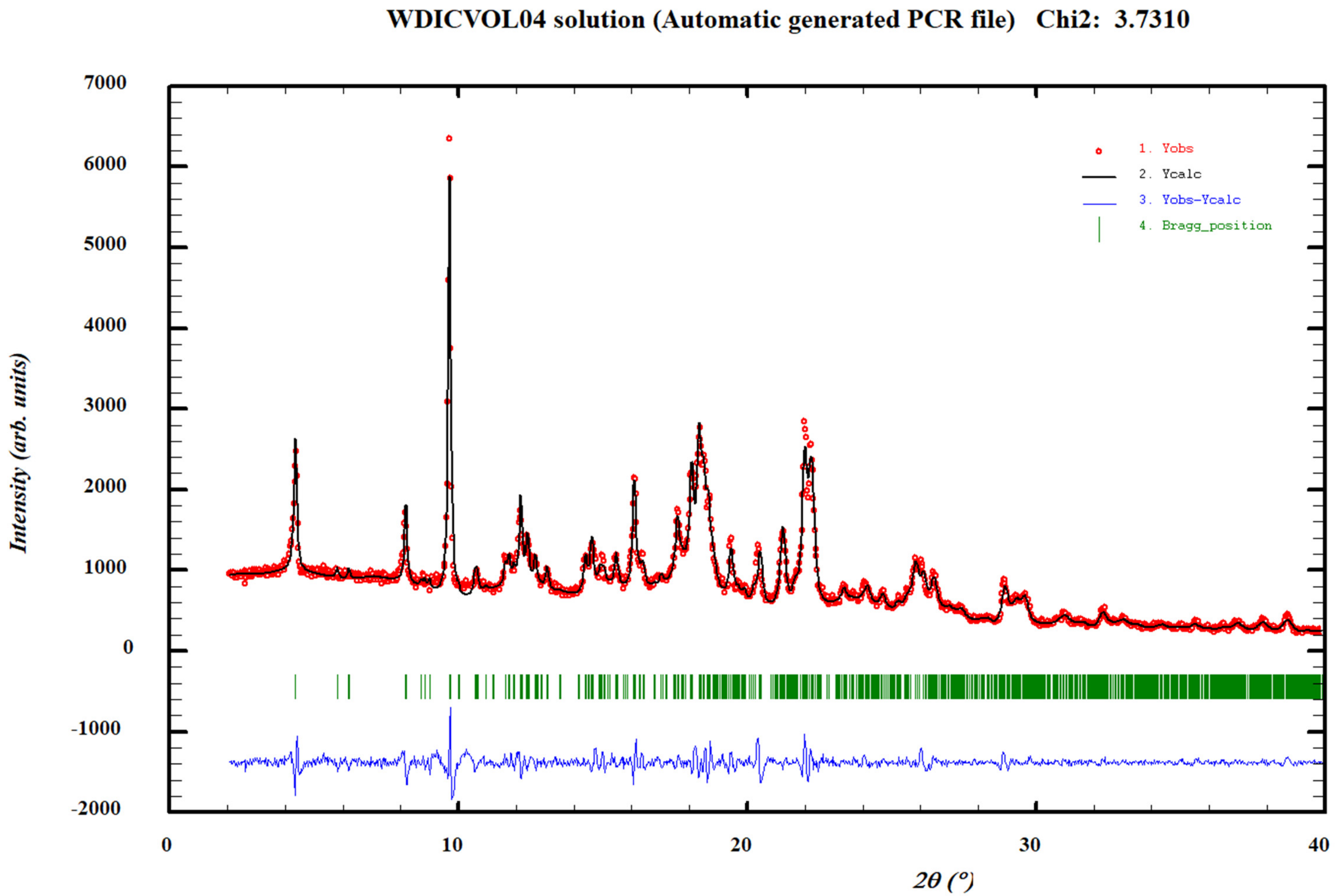

Figure S10. The XRPD of the AD–resorcinol cocrystal (Form III) has been indexed at 298 K with the following proposed triclinic cell: a=29.77(2) Å, b=18.655(9) Å, c=10.021(5) Å, α= 58.24(5)°, β= 134.67(2)°, γ= 101.55(5)° and V=3230(3) Å^3 (Figures of Merit: M_{20}= 10.2, F_{20}= 29.1 (0.0123, 56)), with number of impurities equal to zero. The cell volume is compatible with 4 molecules of adefovir dipivoxil and 4 molecules of resorcinol. (R_{wp}: 6.75; R_{exp}: 3.49), Z=4, (Z value according to estimated density of 1.4 Mg m^{-3}), Z'=2.

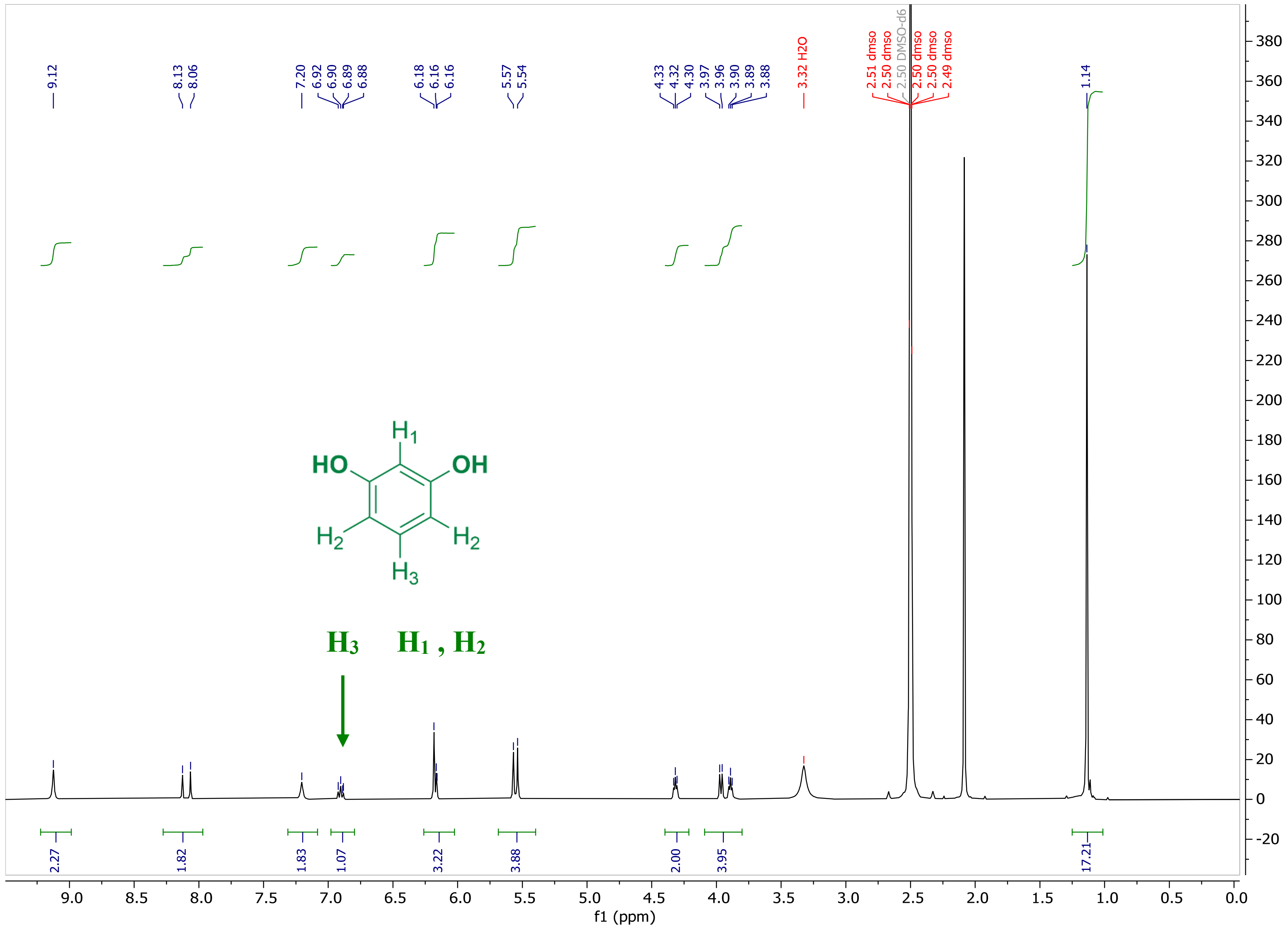

Figure S11. ^{1}H-NMR (dmso-d_6/delay: 1 second /pulse: 45°/scans: 32) of AD–resorcinol cocrystal (Form III) (1:1)

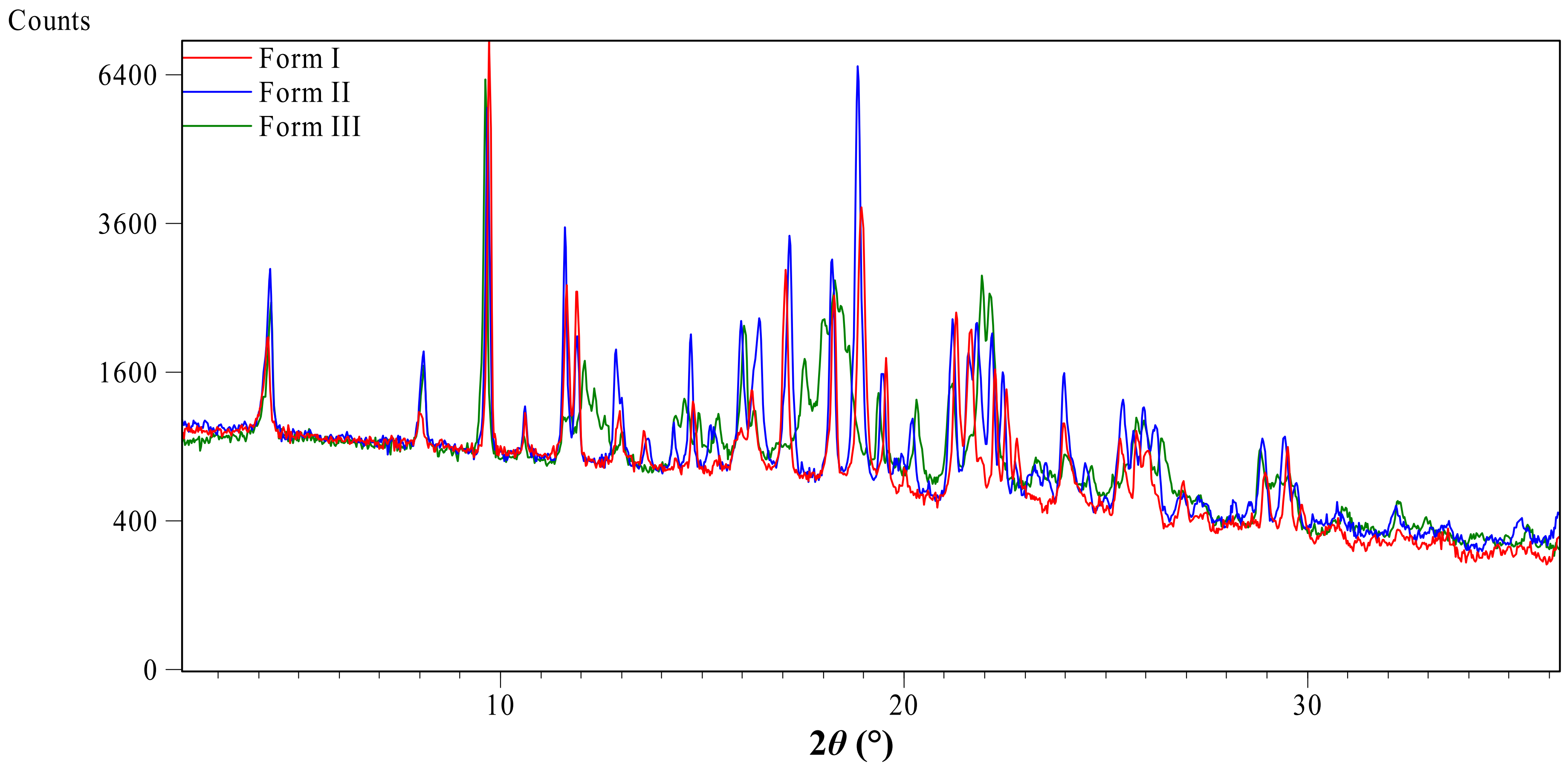

Figure S12. Comparative XRPD diffractograms of the three polymorphs of AD–resorcinol cocrystal: Form I (red), Form II (blue) and Form III (green).

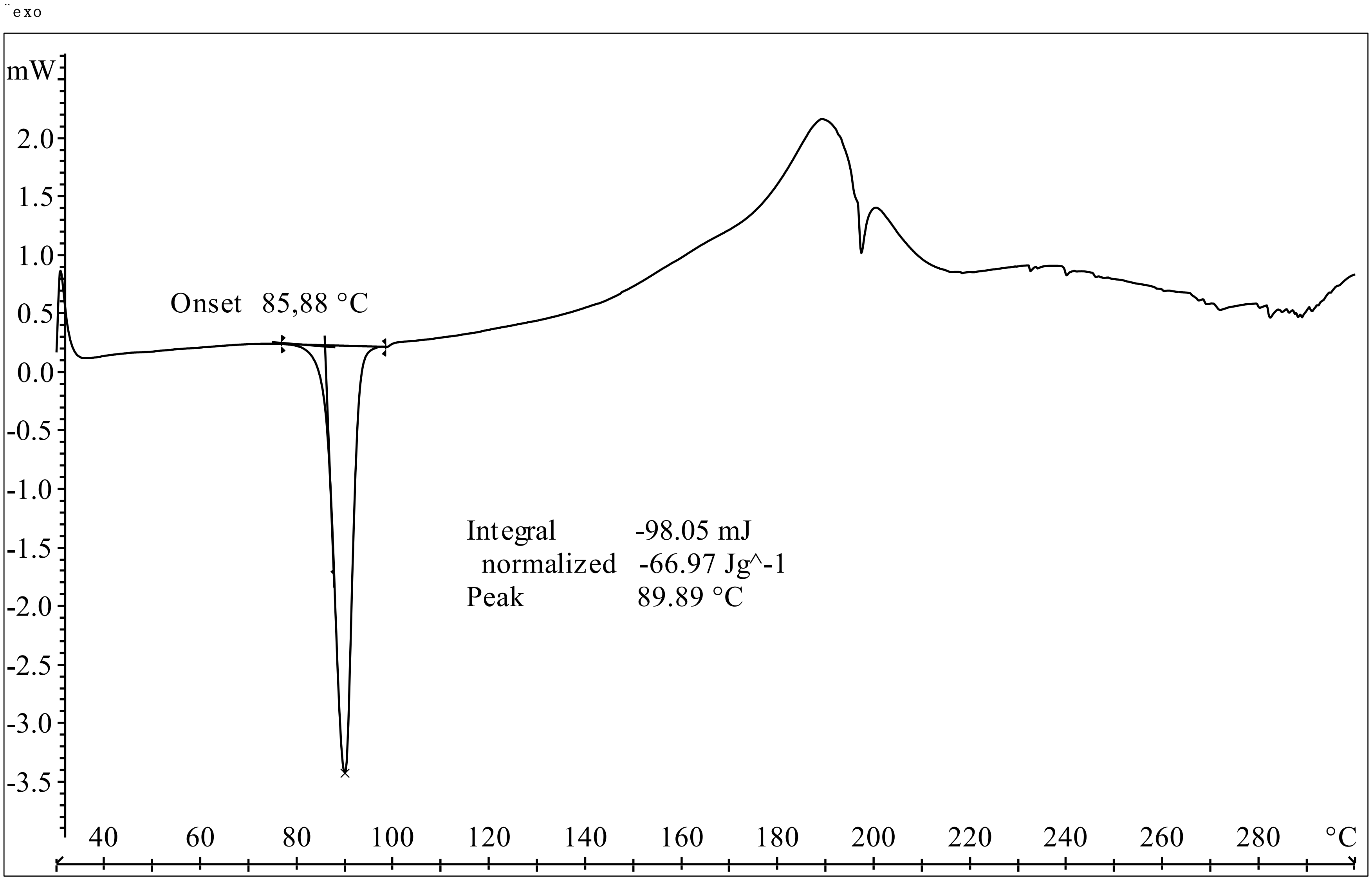

Figure S13. DSC of AD–orcinol cocrystal.

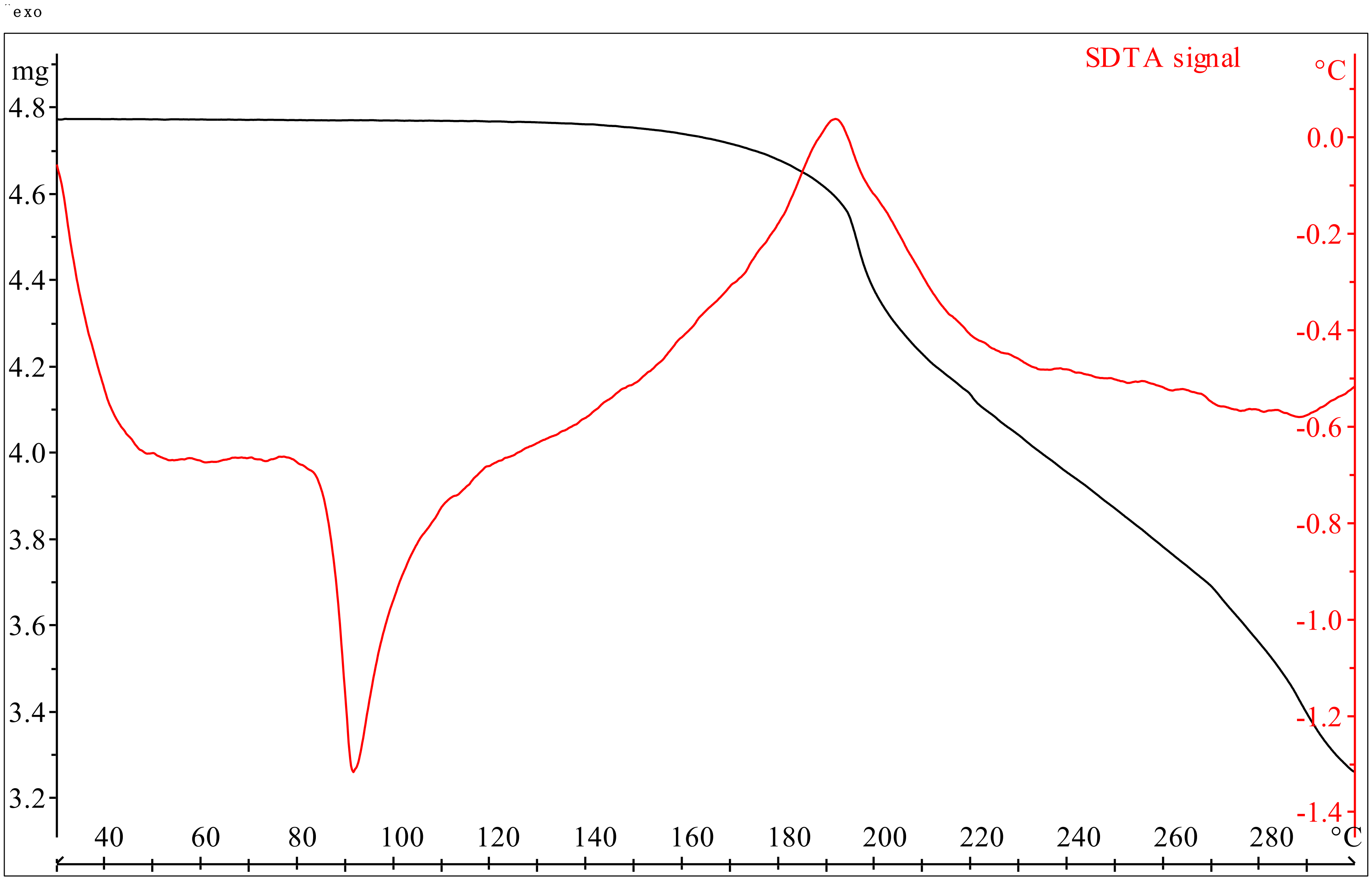

Figure S14. TGA of AD-orcinol cocrystal: the TGA analysis does not show a weight loss before melting.

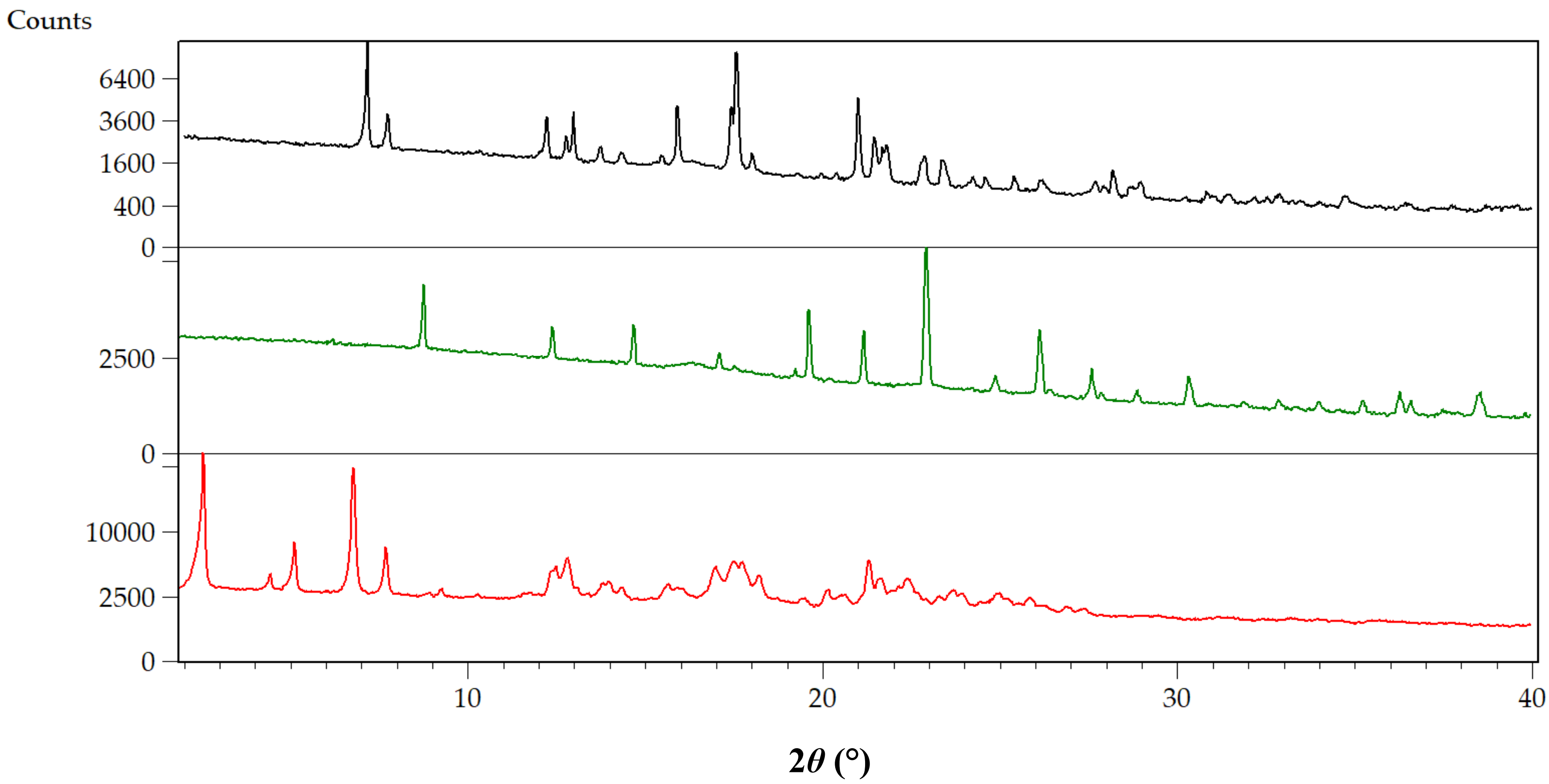

Figure S15. Comparative PXRD diffractograms of adefovir dipivoxil powder used as starting material (black), orcinol (green) and AD-orcinol cocrystal (red).

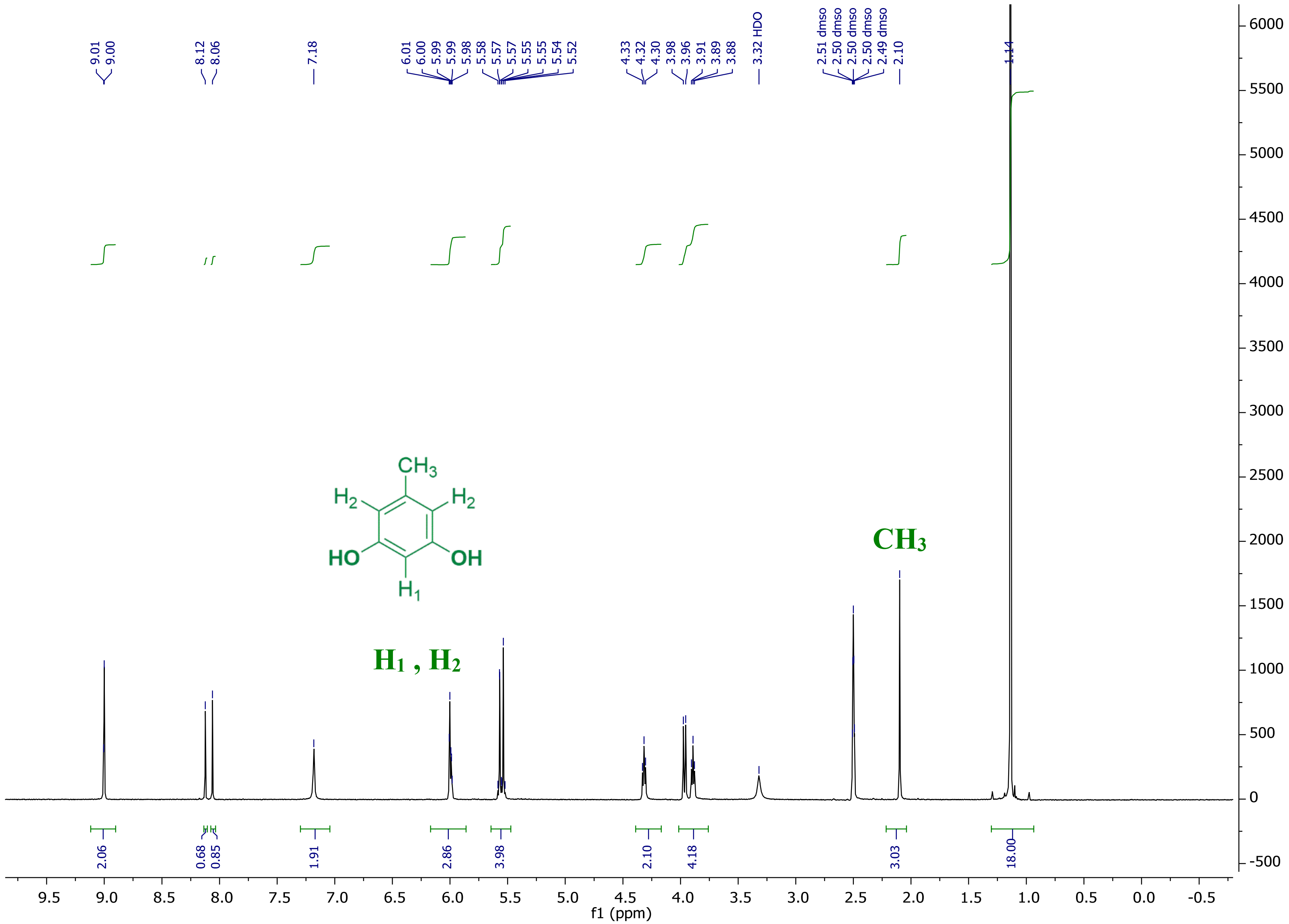

Figure S16. ^{1}H-NMR (dmso-d_6/delay: 1 second /pulse: 45°/scans: 32) of AD–orcinol cocrystal (1:1).

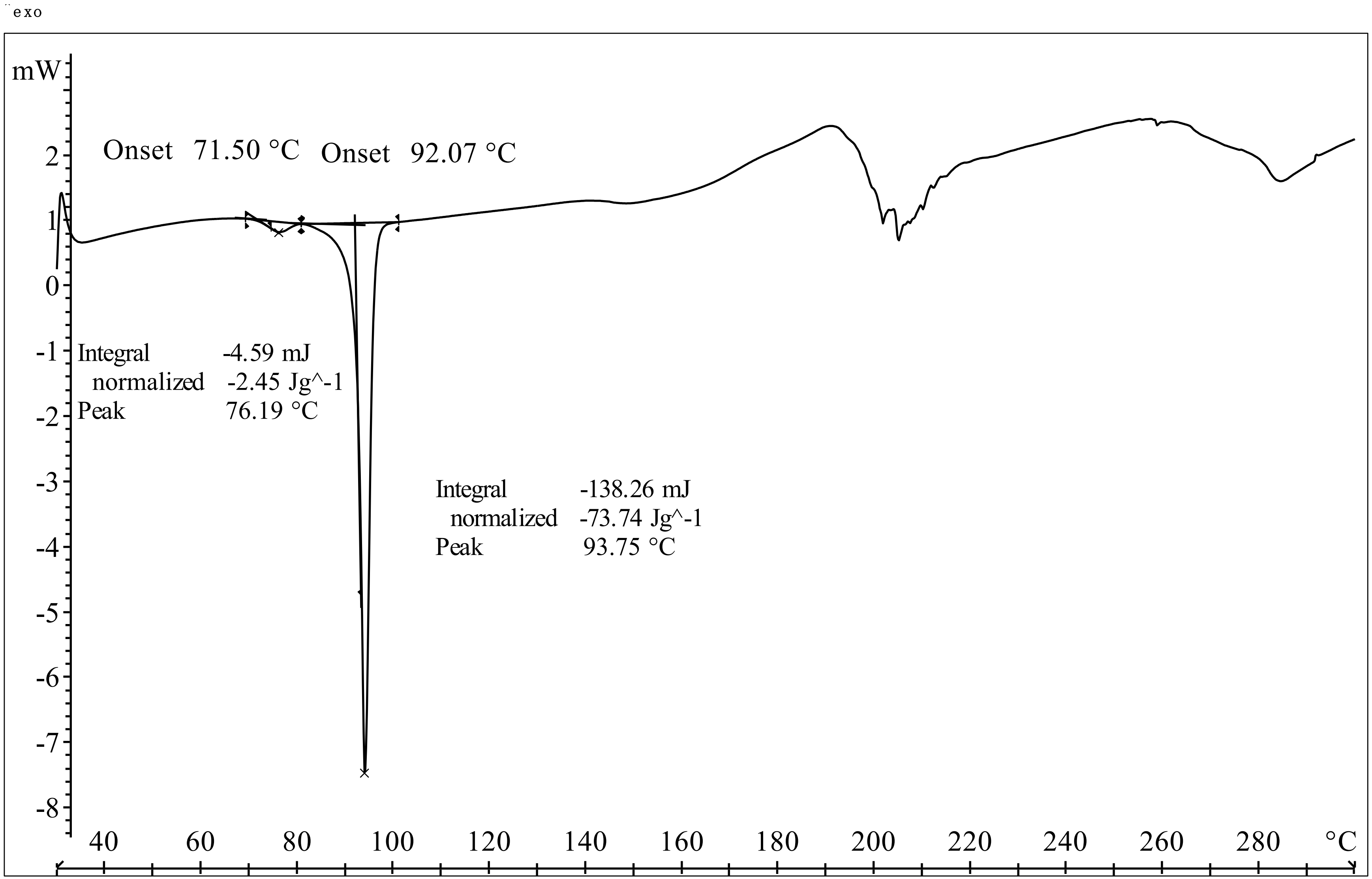

Figure S17. DSC of AD–hydroquinone cocrystal.

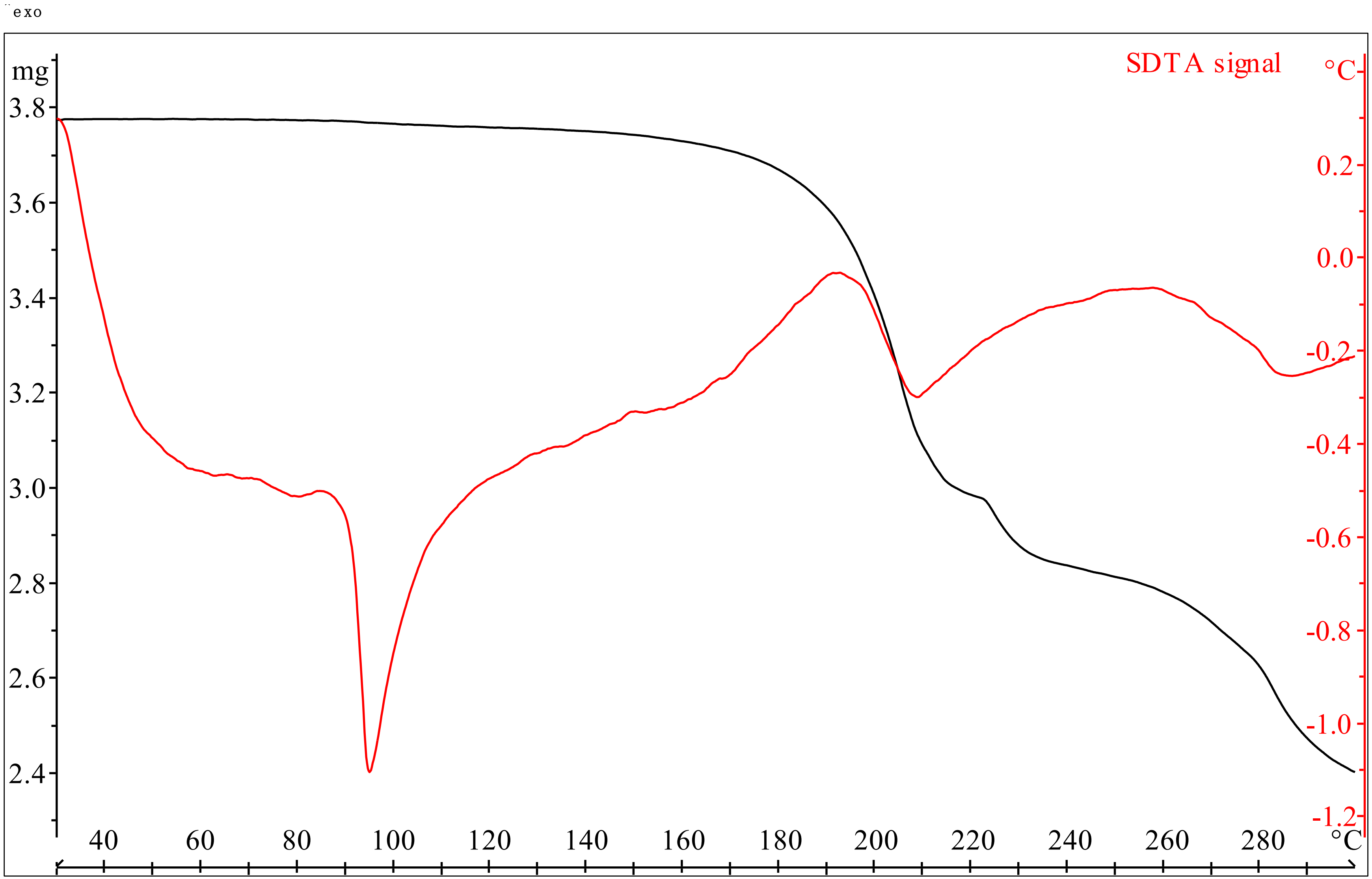

Figure S18. TGA of AD–hydroquinone cocrystal: the TGA analysis does not show a weight loss before melting.

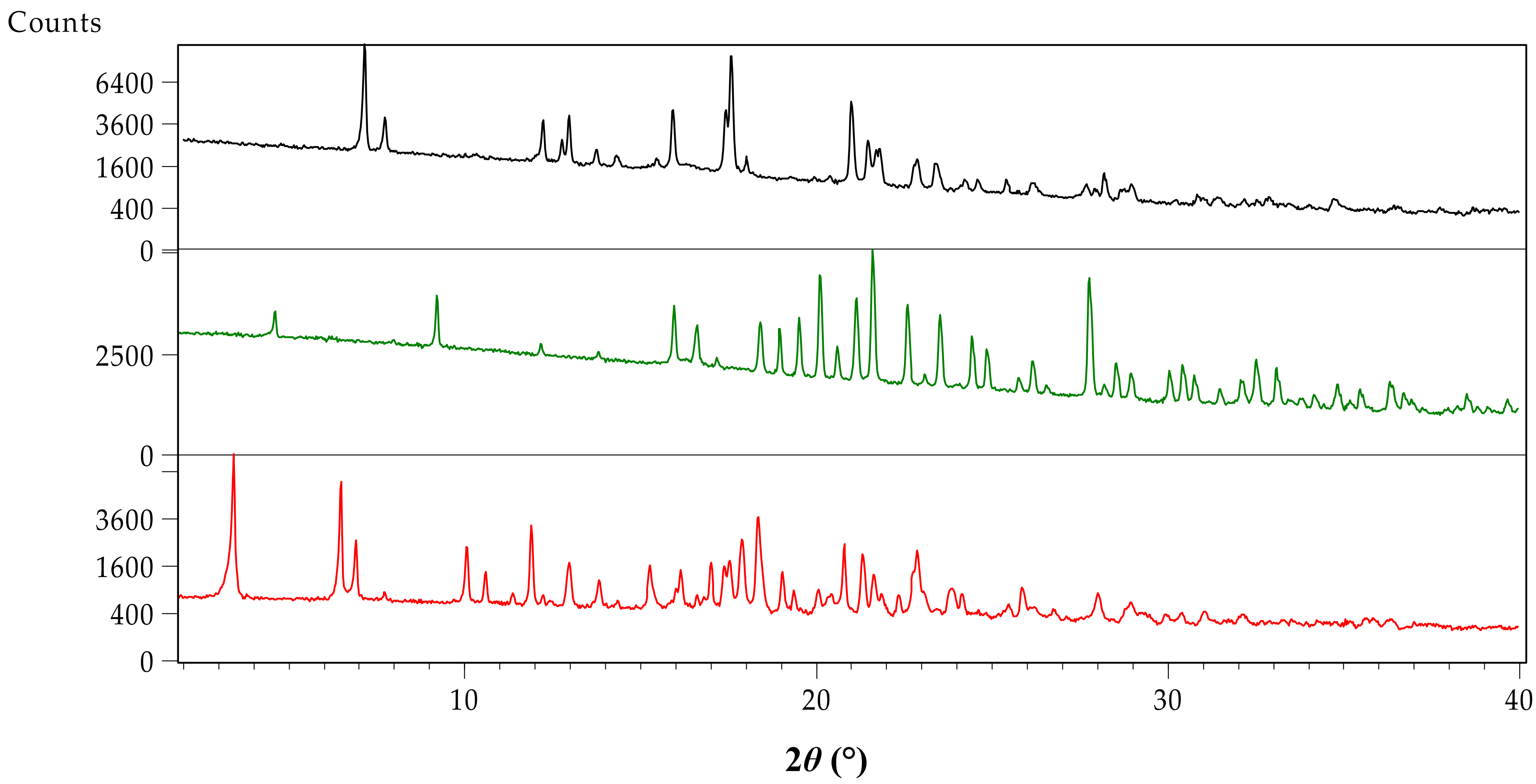

Figure S19. Comparative PXRD diffractograms of adefovir dipivoxil powder used as starting material (black), hydroquinone (green) and AD-hydroquinone cocrystal (red).

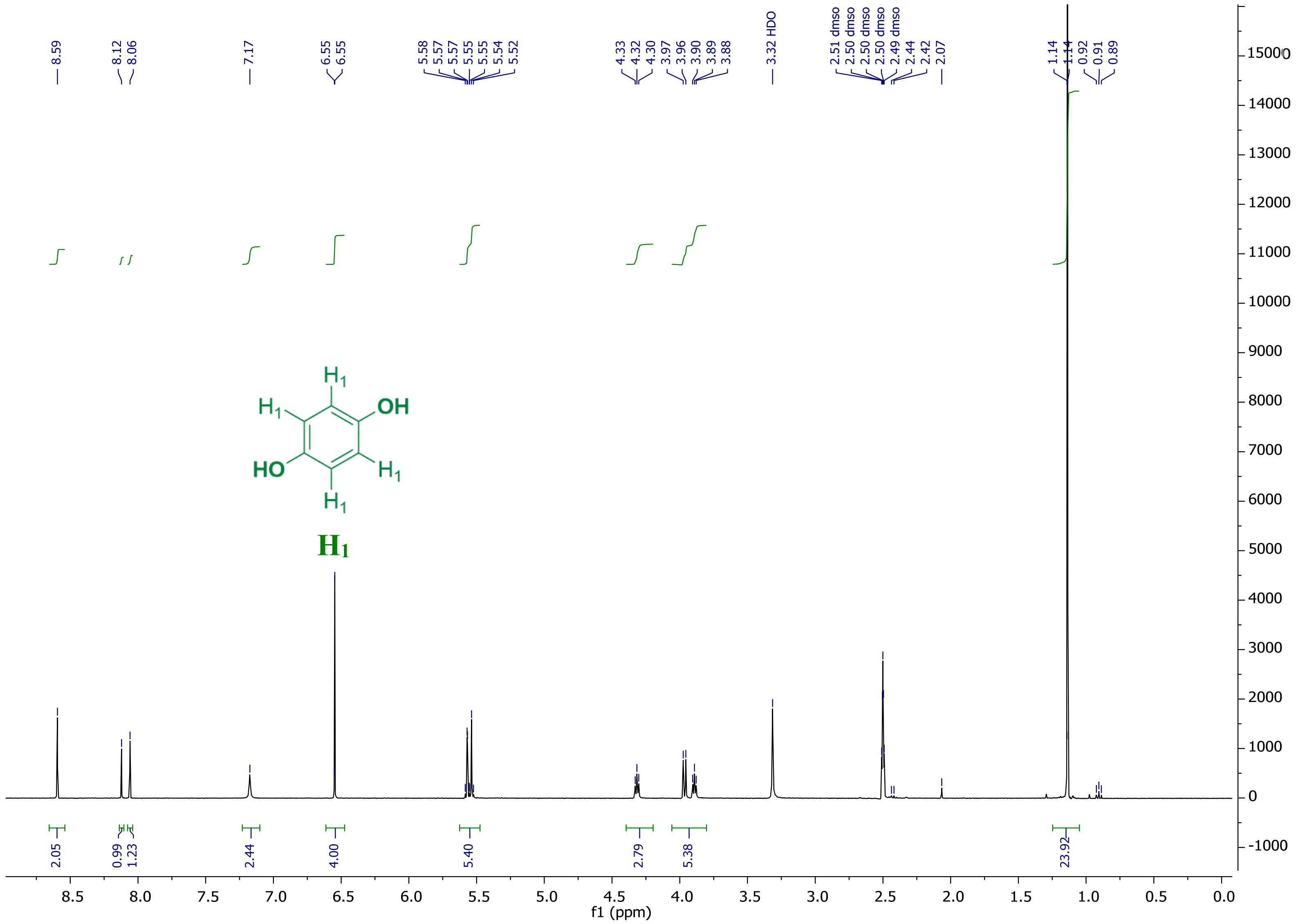

Figure S20. ^{1}H-NMR (dmso-d_6/delay: 1 second /pulse: 45°/scans: 32) of AD–hydroquinone cocrystal (1:1).

5.- Experimental conditions for the dissolution assays.

Table S2. Experimental conditions for the single sector pH dissolution assays. Standard deviation in parenthesis.

Compound	Weight Cocrystal (mg)	Weight API (mg)	pH sector	V_o[a] (mL)	V_f (mL)
AD		8.9 (0.6)	1.96 (0.05)	15	15.46 (0.30)[b]
		9.1 (0.5)	3.88 (0.02)	15	15.29 (0.02)[c]
		9.4 (0.3)	5.46 (0.05)	15	15.59 (0.01)[c]
		9.7 (0.2)	7.40 (0.07)	15	15.93 (0.19)[c]
AD-Res cocrystal (Form I)	7.2 0.5)	5.9 (0.5)	1.94 (0.03)	15	15.28 (0.06)[b]
	7.1 (0.1)	5.8 (0.1)	3.93 (0.01)	15	15.33 (0.01)[c]
	7.1 (0.6)	5.9 (0.5)	5.42 (0.01)	15	15.60 (0.02)[c]
	7.3 (0.3)	6.0 (0.1)	7.37 (0.04)	15	15.96 (0.01)[c]
AD-Orc cocrystal	6.8 (0.6)	5.4 (0.5)	1.95 (0.02)	15	15.25 (0.07)[b]
	7.2 (0.8)	5.8 (0.7)	3.87 (0.06)	15	15.33 (0.01)[c]
	7.5 (0.6)	6.0 (0.5)	5.43 (0.05)	15	15.62 (0.03)[c]
	6.8 (1.5)	5.4 (1.0)	7.38 (0.05)	15	15.95 (0.01)[c]

[a] V_o = 1.5 mL of 0.125 M acetate-phosphate buffer + 13.5 mL 0.15 M KCl; [b] After addition of 0.5 M HCl; [c] After addition of 0.5 M KOH

Table S3. Experimental conditions for the four sector pH dissolution assay. Standard deviation in parenthesis.

Compound	Weight Cocrystal (mg)	Weight API (mg)	pH sector	V_o[a] (mL)	V_f (mL)
AD		9.9	1.53	15	16.00[b]
			3.89		17.29[c]
			5.47		17.60[c]
			7.47		17.94[c]
AD-Res cocrystal (Form I)	7.2 (0.6)	5.9 (0.5)	1.94 (0.03)	15	15.28 (0.06)[b]
			3.89 (0.01)		15.87 (0.10)[c]
			5.41 (0.01)		16.17 (0.10)[c]
			7.34 (0.01)		16.51(0.09)[c]
AD-Orc cocrystal	6.6 (0.7)	5.3 (0.6)	1.94 (0.04)	15	15.27 (0.08)[b]
			3.90 (0.01)		15.87 (0.15)[c]
			5.40 (0.01)		16.15 (0.14)[c]
			7.30 (0.01)		16.49 (0.14)[c]

[a] V_o = 1.5 mL of 0.125 M acetate-phosphate buffer + 13.5 mL 0.15 M KCl; [b] After addition of 0.5 M HCl; [c] After addition of 0.5 M KOH

Table S4. Experimental conditions for the four sector pH biphasic-dissolution assays. Standard deviation in parenthesis.

Compound	Weight Cocrystal (mg)	Weight API (mg)	pH sector	V_o – aq[a] (mL)	V_f – aq (mL)	V_d - dec[d] (mL)
AD		9.8 (0.2)	1.53 (0.01)	40	43.78 (0.06)[b]	30
			4.05 (0.01)		47.22 (0.01)[c]	
			5.46 (0.01)		47.86 (0.01)[c]	
			7.37 (0.01)		48.70 (0.01)[c]	
AD-Res cocrystal (Form I)	10.8 (1.2)	8.8 (1.0)	1.53 (0.01)	40	43.69 (0.06)[b]	30
			4.05 (0.01)		47.05 (0.15)[c]	
			5.46 (0.01)		47.68 (0.16)[c]	
			7.37 (0.01)		48.55 (0.26)[c]	
AD-Orc cocrystal	10.4 (1.7)	8.6 (1.4)	1.53 (0.01)	40	43.75 (0.04)[b]	30
			4.05 (0.01)		47.19 (0.09)[c]	
			5.48 (0.01)		47.82 (0.08)[c]	
			7.39 (0.01)		48.61 (0.08)[c]	
AD-Hyd cocrystal	11.27 (0.1)	9.24 (0.1)	1.55 (0.01)	40	43.70 (0.14)[b]	30
			4.05 (0.01)		46.87 (0.16)[c]	
			5.46 (0.01)		47.51 (0.16)[c]	
			7.37 (0.01)		48.33 (0.18)[c]	

[a] V_o = 4 mL of 0.125M acetate-phosphate buffer + 36 mL 0.5 M KCl; [b] After addition of0.5 M HCl; [c] After addition of 0.5 M KOH; [d] volume of decanol added in the second sector.

6.- Determination of the Molar Extinction Coefficients and acidity constants.

Molar extinction coefficients (MEC) of Adefovir dipivoxyl and the coformers were determined by UV-metric titration using a GlpKa™ titrator (Sirius Analytical Instruments, UK). 10 mM stock solution of each compound was prepared in DMSO. 50 µL of sample stock solution and 0.25 mL of 15 mM potassium phosphate buffer were added to 10 mL of 0.15 M KCl solution. The pH was adjusted to 2 with 0.5 M HCl before starting the titration, and then the titration was done using 0.5 M KOH up to pH 12. The UV absorption spectra (between 250 and 400 nm) of the solution were recorded at each titrant addition by a fiber optic dip-probe. The collected data were refined through the inForm Control and Assay Designer software (version 1.6.0.0), and Molar Extinction Coefficients (Figure S21) and the pK_a values (Table S5) obtained by Target Factor Analysis. The plot shows that molar absorption of the two species of AD is much higher than that of the cocrystals (for the cocrystals only the profile of the neutral species is shown, as it is the species present at all working pH values). It can be observed the total overlapping between the spectra of AD and the one of resorcinol and orcinol in the studied wavelength range. Hydroquinone, however, has an extra signal starting at 288 nm, so that at wavelengths higher than 300 nm, its contribution can be distinguished from the one of adefovir.

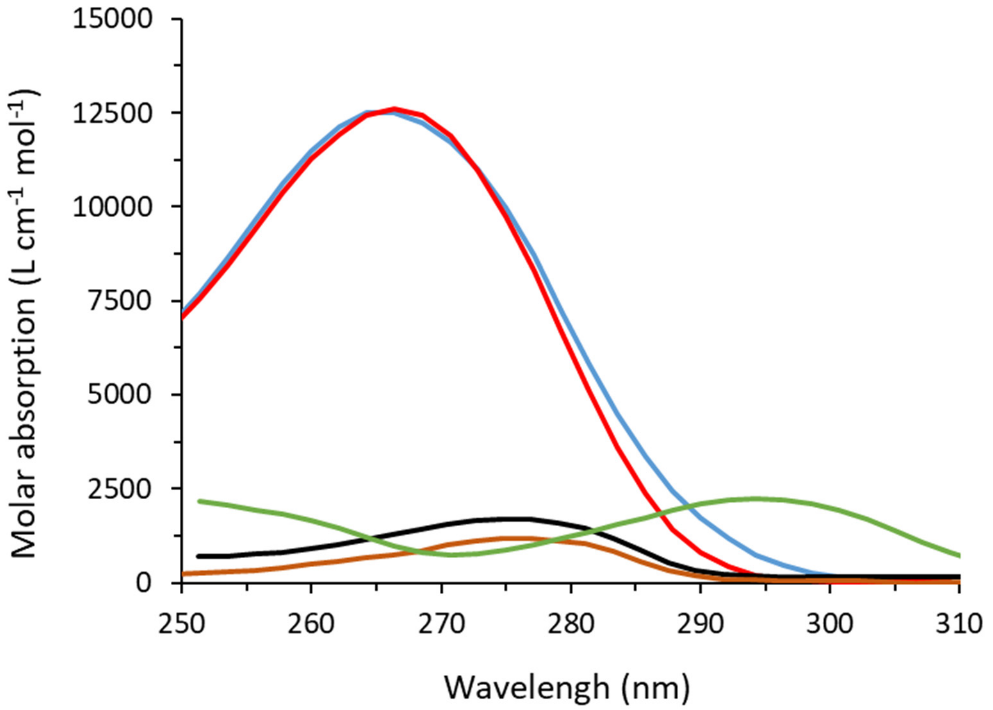

Figure S21. MEC absorption profiles of the different species of Adefovir dipivoxyl and the neutral species of the coformers: HAD^{+} (—); AD (—); Resorcinol (—); Orcinol (—); Hydroquinone (—).

Table S5. pK_a values of the compounds under study at 25 ºC and zero ionic strength.

Name	Type of compound	pK_{a1}	pK_{a2}
Adefovir dipivoxil	BH+	3.78 (0.05)	
Resorcinol	H2A	9.46 (0.02)	11.61 (0.03)
Orcinol	H2A	9.60 (0.02)	11.75 (0.02)
Hydroquinone	H2A	10.20 (0.02)	---
